# Graded Ca^2+^/calmodulin-dependent coupling of voltage-gated Ca_V_1.2 channels

**DOI:** 10.7554/eLife.05608

**Published:** 2015-02-25

**Authors:** Rose E Dixon, Claudia M Moreno, Can Yuan, Ximena Opitz-Araya, Marc D Binder, Manuel F Navedo, Luis F Santana

**Affiliations:** 1 Department of Physiology and Biophysics, University of Washington, Seattle, United States; 2 Department of Pharmacology, University of California, Davis, Davis, United States; The University of Texas at Austin, United States

**Keywords:** coupled gating, calcium sparklets, EC coupling, calmodulin, voltage-gated calcium channels, mouse

## Abstract

In the heart, reliable activation of Ca^2+^ release from the sarcoplasmic reticulum during the plateau of the ventricular action potential requires synchronous opening of multiple Ca_V_1.2 channels. Yet the mechanisms that coordinate this simultaneous opening during every heartbeat are unclear. Here, we demonstrate that Ca_V_1.2 channels form clusters that undergo dynamic, reciprocal, allosteric interactions. This ‘functional coupling’ facilitates Ca^2+^ influx by increasing activation of adjoined channels and occurs through C-terminal-to-C-terminal interactions. These interactions are initiated by binding of incoming Ca^2+^ to calmodulin (CaM) and proceed through Ca^2+^/CaM binding to the Ca_V_1.2 pre-IQ domain. Coupling fades as [Ca^2+^]_i_ decreases, but persists longer than the current that evoked it, providing evidence for ‘molecular memory’. Our findings suggest a model for Ca_V_1.2 channel gating and Ca^2+^-influx amplification that unifies diverse observations about Ca^2+^ signaling in the heart, and challenges the long-held view that voltage-gated channels open and close independently.

**DOI:** http://dx.doi.org/10.7554/eLife.05608.001

## eLife digest

To pump blood around the body, the muscle cells within the heart must contract and relax together with a regular rhythm. A contraction begins when proteins called Ca_V_1.2 channels embedded in the cell membranes of heart cells open to allow calcium ions to enter the cells. The calcium ions that enter through these Ca_V_1.2 channels trigger the release of calcium ions from storage compartments within the cells, which leads to the heart contracting. However, to trigger this release of calcium ions, many Ca_V_1.2 channels have to open at the same time and we do not yet know how this is co-ordinated.

Dixon et al. studied Ca_V_1.2 channels in heart muscle cells from mice. The experiments show that these proteins are arranged in clusters of eight, on average, in the cell membrane. When calcium ions enter the cell they bind to a protein called calmodulin, which in turn binds to a Ca_V_1.2 channel. This allows the Ca_V_1.2 channels within a cluster to interact with each other. The physical association between Ca_V_1.2 channels within clusters enables them to work cooperatively; they open at the same time to allow more calcium ions to enter and then close together to allow the cell to relax.

Dixon et al. found that even when levels of calcium ions in the cells declined, the Ca_V_1.2 channels within clusters remained open for a little while longer before they closed. This suggests that the interactions between the Ca_V_1.2 channels act as a type of ‘molecular memory’ that may alter how the cells respond to future activity.

These results challenge the previously held view that the Ca_V_1.2 channels open and close independently of one another. Future studies will seek to understand the molecular details of how these channels cluster together, and how this clustering affects changes in heart rate and heart abnormalities like long QT syndrome.

**DOI:** http://dx.doi.org/10.7554/eLife.05608.002

## Introduction

L-type Ca^2+^ channels are composed of a pore-forming α_1_ subunit and four additional accessory subunits (α_2_, β, γ, δ) (Catterall, 1995). Four different α_1_ subunits have been identified to date, one of which, Ca_V_1.2, is expressed in neurons as well as cardiac and arterial smooth muscle (Koch et al., 1990; Navedo et al., 2007; Zhang et al., 2007). Four distinct genes encode L-type Ca^2+^ channel β-subunits, each with multiple splice variants. In addition, four α_2_δ genes have been identified. Both cell- and tissue-specific combinations of these Ca_V_1.2 subunits endow the channels with distinct functional properties (Catterall, 2000).

A prominent characteristic of Ca_V_1.2 channels is the tight regulation of their activity by the Ca^2+^ signals they produce (Ben-Johny and Yue, 2014). For example, increases in [Ca^2+^]_i_ have been implicated in Ca_V_1.2 facilitation; this Ca^2+^-dependent facilitation (CDF) is a form of positive feedback that amplifies Ca^2+^ influx. An increase in intracellular Ca^2+^ concentration ([Ca^2+^]_i_) has also been proposed to exert the opposite effect—Ca^2+^-dependent inactivation (CDI). Thus, the balance between CDF and CDI of Ca_V_1.2 channels plays a key role in regulating the magnitude of Ca^2+^ influx. The general consensus is that CDF and CDI involve Ca^2+^ binding to calmodulin (CaM) in the IQ domain in the C-terminal tail of these channels.

During excitation-contraction (EC) coupling, membrane depolarization opens Ca_V_1.2 channels in the sarcolemma of ventricular myocytes. This allows a small amount of Ca^2+^ to enter ventricular myocytes that can be detected optically in the form of a ‘Ca_V_1.2 sparklet’, raising local [Ca^2+^]_i_ beyond the threshold for activation of ryanodine receptors (RyRs) in the sarcoplasmic reticulum (Wang et al., 2001). Synchronous activation of multiple RyRs by Ca_V_1.2 channels produces a global rise in [Ca^2+^]_i_ that initiates myocardial contraction (Cheng et al., 1993).

EC coupling in ventricular myocytes is remarkably reproducible, with each action potential (AP) invariably evoking a whole-cell [Ca^2+^]_i_ transient that results in contraction. At the membrane potentials reached during the plateau of the ventricular AP (approximately +50 mV), the driving force for Ca^2+^ entry at physiological Ca^2+^ levels (∼2 mM) is so low that opening of a single Ca_V_1.2 channel is not sufficient to raise local [Ca^2+^]_i_ beyond the RyR activation threshold. However, the probability of RyR activation during this phase of the AP is very high (>0.9). This degree of reliability would presumably require 5–10 Ca_V_1.2 channels to open simultaneously (Inoue and Bridge, 2003; Sobie and Ramay, 2009). However, because the maximum open probability (*P*_o_) of Ca_V_1.2 channels at physiological [Ca^2+^]_o_ is ∼0.3 (Josephson et al., 2010), the probability of 5–10 independently gating channels opening simultaneously is extremely low (i.e., 0.3^5^ to 0.3^10^). This raises a fundamental question: if the probability of coincident openings of the requisite number of Ca_V_1.2 channels is so low, why is the probability of RyR activation during the cardiac AP so high? Answering this question is critical for understanding the mechanistic basis of reliable cardiac performance.

A potential answer to this conundrum lies in the recently proposed concept that clusters of Ca_v_1.2 channels can be functionally coupled to one another through physical interactions between their C-terminal tails (Dixon et al., 2012). This interaction enables physically linked channels to coordinate their gating, leading to amplification of Ca^2+^ influx, Ca^2+^ current facilitation, and EC coupling in ventricular myocytes. Importantly, this model challenges the long-held assumption that Ca_v_1.2 channels, like other classes of voltage-gated channels, function exclusively as monomers that gate independently of one another. To date, however, the mechanism underlying functional Ca_V_1.2 channel coupling has remained unknown.

Here, using a combination of super-resolution nanoscopy, Ca^2+^-imaging, electrophysiology and two methodologically distinct assays of protein–protein interaction, we have assembled a body of evidence that significantly alters our current understanding of Ca^2+^/CaM regulation of Ca_V_1.2 channels and reconciles diverse observations about Ca^2+^ signaling in ventricular myocytes. We discovered that binding of Ca^2+^ to CaM induces C-terminal-to-C-terminal Ca_V_1.2 channel interactions that increase the activity of adjoined channels, facilitating Ca^2+^ currents and increasing Ca^2+^ influx. Notably, we found that functional coupling outlasts the Ca^2+^ current that evokes it, providing evidence for a type of ‘molecular memory’ that could transiently shape the response of the cell to subsequent APs. We propose that cooperative gating of Ca_V_1.2 channels is a new general mechanism for the regulation of excitability and Ca^2+^ influx in cardiac myocytes and suggest that this concept can be extended to other excitable cells.

## Results

### Ca^2+^ ions augment functional coupling of Ca_V_1.2 channels

**Figure 1.**
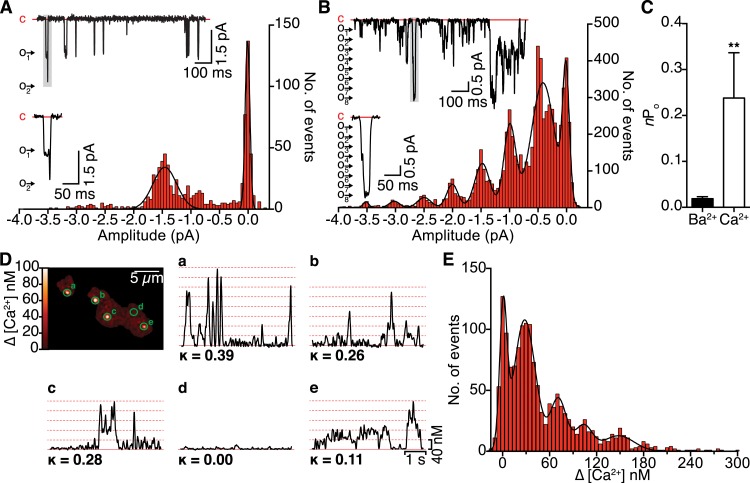
Single-channel electrical and optical recordings of Ca_V_1.2 channel coupling. (**A** and **B**) Representative *i*_Ba_ (**A**) and *i*_Ca_ (**B**) traces recorded from Ca_V_1.2-expressing tsA-201 cells during step depolarizations from −80 to −30 mV. Amplitude histograms (constructed from *n* = 6 cells each) were fit with multi-component Gaussian functions (solid black lines). A portion of each trace (gray box) is shown enlarged below, showing that the resulting L-type Ca^2+^ current reflects the simultaneous opening and closing of multiple channels with Ca^2+^ as the charge carrier, but not with Ba^2+^ as the charge carrier. (**C**) Bar chart of *i*_Ba_ and *i*_Ca_ single-channel activity (*n*P_o_). Data are presented as means ± SEM (**p < 0.01). (**D**) Calibrated TIRF image of an adult ventricular myocyte dialyzed with the Ca^2+^ indicator dye Rhod-2 via the patch pipette (see also Video 1). Time courses of [Ca^2+^]_i_ from each sparklet site (indicated by green circles on TIRF image) and their κ values are shown in panels **a**-**e**. (**E**) All-points histogram of Ca^2+^ sparklet data recorded from adult ventricular myocytes. The data were fit with a multi-component Gaussian function (solid black line). **DOI:** http://dx.doi.org/10.7554/eLife.05608.003

We began our study by expressing Ca_V_1.2 channels in tsA-201 cells and recording elementary Ca_V_1.2 currents from cell-attached patches. Currents were elicited with a step depolarization to −30 mV with Ba^2+^ or Ca^2+^ as the charge carrier (Figure 1A,B). The mean amplitudes of i_Ca_ and i_Ba_ were 0.50 ± 0.02 (*n* = 6) and 1.45 ± 0.01 pA (*n* = 6), respectively. All-points histograms revealed that multi-channel openings were more likely with Ca^2+^ than with Ba^2+^. Accordingly, the activity (*n*P_o_), defined as the number of channels (*n*) times the open probability (P_o_), of Ca_V_1.2 channels within a patch, was significantly higher with Ca^2+^ (0.24 ± 0.10) than Ba^2+^ (0.02 ± 0.01; Figure 1C). Closer inspection of the multi-channel openings revealed that, with Ca^2+^ as the charge carrier, multiple channels frequently opened together instantaneously and subsequently closed together. For example, in the enlarged trace in Figure 1B, four channels opened simultaneously, followed by the opening of four additional channels. The eight channels remained open for a time (∼11.5 ms), then all closed simultaneously. This apparent coordinate gating of multiple Ca_V_1.2 channels was not observed when Ba^2+^ was used as the charge carrier. These results challenge the long-held and generally accepted view that individual Ca_V_1.2 channels gate independently, and instead strongly suggest that these channels frequently exhibit ‘cooperative gating’. An additional implication is that Ca^2+^ itself increases the probability of cooperative Ca_V_1.2 channel gating.

To test whether this hypothesis holds true in primary cells, we recorded Ca_V_1.2 Ca^2+^ sparklet activity in freshly isolated adult ventricular myocytes (Figure 1D and Video 1). A multi-Gaussian fit to the all-points histogram of the calibrated Ca^2+^ signal obtained with 20 mM [Ca^2+^]_o_ revealed quantal amplitudes of 36.8 ± 4.2 nM (Figure 1E), in excellent agreement with the ∼38 nM reported previously for Ca_V_1.2 Ca^2+^ sparklets in arterial smooth muscle cells and tsA-201 cells (Navedo et al., 2005, 2006). Consistent with our single-channel data, we frequently observed multi-quantal Ca^2+^ sparklets corresponding to the simultaneous opening of several Ca_V_1.2 channels. We next applied a coupled Markov chain model to determine whether these Ca^2+^-influx events were solely attributable to stochastic, independently gating Ca_V_1.2 channels or instead reflected cooperative channel gating. The model assigns a coupling coefficient (κ) value to each Ca^2+^ sparklet site, ranging from 0 for independently gating channels to 1 for channels that gate exclusively in a cooperative manner (Chung and Kennedy, 1996; Navedo et al., 2010). Using κ > 0.1 as a threshold for cooperative gating (Navedo et al., 2010), we found that the majority of Ca^2+^ sparklet sites displayed cooperative or ‘coupled’ gating behavior, consistent with our hypothesis. Together, these data suggest that functional coupling of Ca_V_1.2 channels is a Ca^2+^-dependent phenomenon.

**Video 1.** Cardiomyocyte Ca^2+^ sparklets. Stack of 2D images acquired at 100 Hz from a whole-cell patch-clamped adult ventricular myocyte held at −80 mV and dialyzed with Rhod-2 via the patch pipette. **DOI:** http://dx.doi.org/10.7554/eLife.05608.004

### Ca_V_1.2 channels form clusters in ventricular myocytes

**Figure 2.**
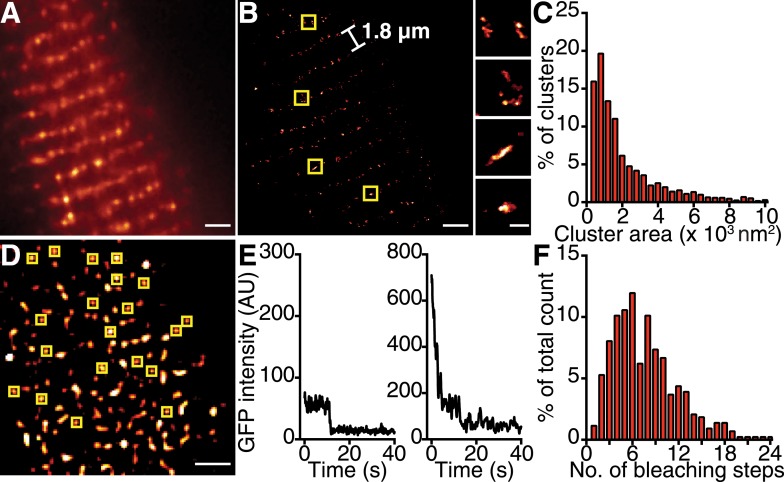
Ca_V_1.2 channels form clusters in the ventricular myocyte PM. (**A**) TIRF image of a fixed, adult mouse ventricular myocyte immunolabeled with an antibody specific for Ca_V_1.2 channels. (**B**) Super-resolution GSD image of the same cell. Channels are located along the t-tubule network with the characteristic 1.8-μm separation. Yellow boxes denote location of higher-magnification images of channel clusters (*right*). (**C**) Distribution of cluster areas in ventricular myocytes (*n* = 5 myocytes). (**D**) An average of the first five frames of a TIRF image time series taken of a myocyte isolated from mice expressing Ca_V_β_2a_-PA-GFP. Yellow boxes indicate spots selected for analysis. Scale bars = 2 μm. (**E**) Examples of bleaching steps for Ca_V_β_2a_-PA-GFP associated with Ca_V_1.2 channels. (**F**) Distribution of bleaching steps obtained from 435 spots selected from *n* = 11 cells. **DOI:** http://dx.doi.org/10.7554/eLife.05608.005

**Figure 3.**
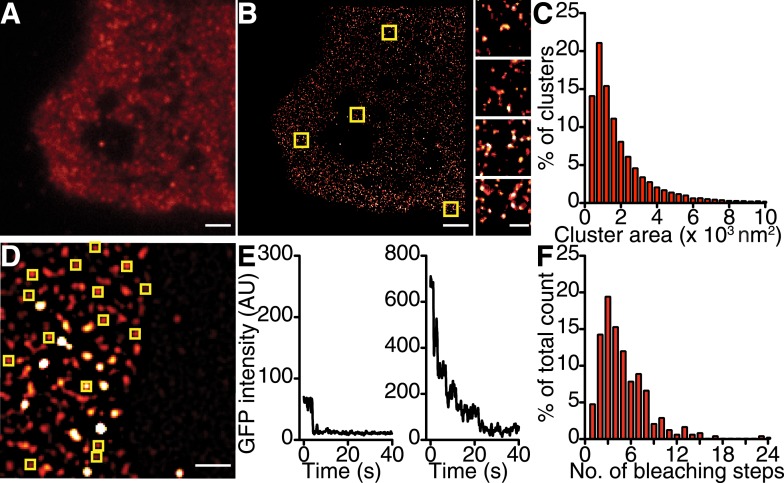
Ca_V_1.2 channels form clusters in tsA-201 cell membranes. (**A** and **B**) TIRF and GSD images of immunolabeled Ca_V_1.2 channels in a transfected tsA-201 cell (**A**). Yellow boxes in (**B**) indicate the location of each higher-magnification image (*right*). (**C**) Distribution of cluster areas in tsA-201 cells (*n* = 9 cells). (**D**) An average of the first five frames of a TIRF image time series for a tsA-201 cell expressing Ca_V_1.2-EGFP. (**E**) Examples of bleaching steps for Ca_V_1.2-EGFP. Scale bars = 2 μm. (**F**) Distribution of bleaching steps obtained from 484 spots selected from *n* = 10 cells. **DOI:** http://dx.doi.org/10.7554/eLife.05608.006

If the trigger for Ca_V_1.2 channel coupling were a local elevation in [Ca^2+^]_i_ resulting from the opening of a single channel, then the efficacy of this signal in recruiting a nearby channel would be critically dependent on the distance separating the channels. Using super-resolution nanoscopy (Folling et al., 2008), we examined the spatial organization of endogenous Ca_V_1.2 channels in ventricular myocytes (Figure 2A–C) and heterologously expressed Ca_V_1.2 channels in tsA-201 cells (Figure 3A–C). Our data clearly show that Ca_V_1.2 channels formed clusters along the sarcolemmal Z-lines of ventricular myocytes (Figure 2A,B) and throughout the plasma membrane (PM) of tsA-201 cells (Figure 3A,B). The average area occupied by a Ca_V_1.2 channel cluster was 2555 ± 82 nm^2^ in ventricular myocytes (*n* = 5; Figure 2C) and 2190 ± 20 nm^2^ in tsA-201 cells (*n* = 9; Figure 3C).

**Figure 3—figure supplement 1.**
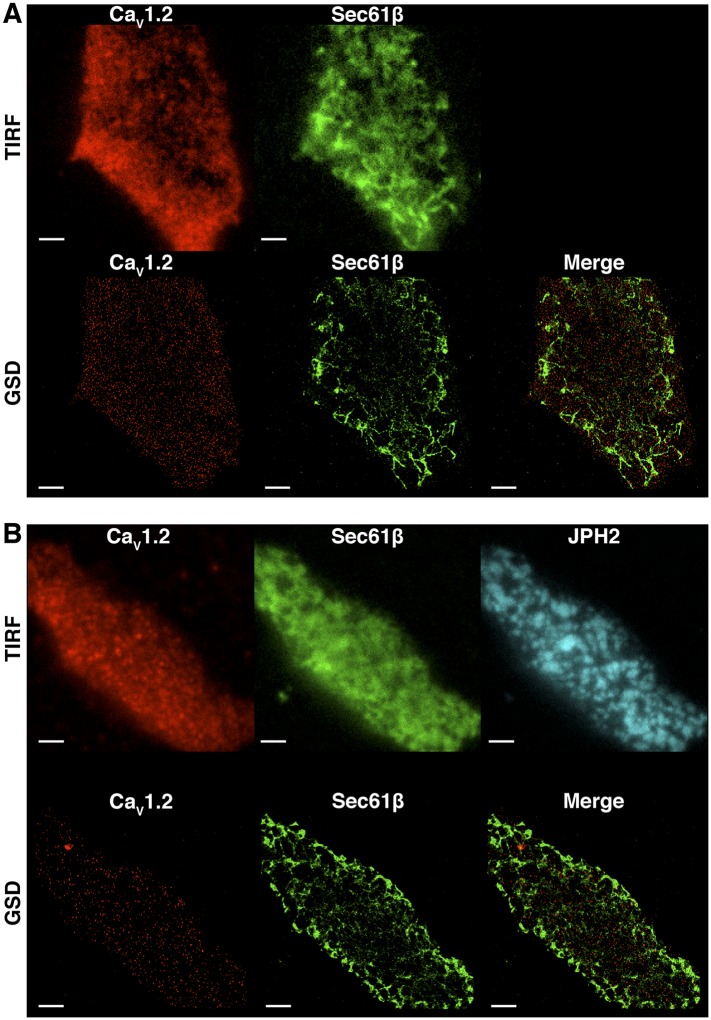
Ca_V_1.2 channel clusters are not co-localized to tsA-201 ER structures. (**A**) TIRF (*top*) and GSD (*bottom*) images of immunolabeled Ca_V_1.2 channels (*left*) and mCherry-Sec61β (*middle*) in a transfected tsA-201 cell. The image on the bottom right was generated by merging Ca_V_1.2 and mCherry-Sec61β GSD images. (**B**) *Top*: TIRF images of immunolabeled Ca_V_1.2 channels (*left*), mCherry-Sec61β (*middle*), and JPH2 (*right*) in a transfected tsA-201 cell. *Bottom*: GSD images from the same cell showing Ca_V_1.2 channels (*left*), mCherry-Sec61β (*middle*), and a merge of the two (*right*). Scale bars = 2 μm. **DOI:** http://dx.doi.org/10.7554/eLife.05608.007

Since Ca_V_1.2 channel clusters were localized to the t-tubule regions of ventricular myocytes where the junctional SR (jSR) comes into close apposition to the myocyte PM, one might predict that the channel clusters would similarly localize to PM-adjacent ER structures in tsA-201 cells. To test this idea, we co-expressed mCherry-sec61β (a general ER marker (Zurek et al., 2011)) in tsA-201 cells together with Ca_V_1.2 channels. Super-resolution imaging revealed that Ca_V_1.2 channel cluster distribution was not restricted to regions of the surface membrane where the ER was located (Figure 3—figure supplement 1A). Instead, these channels were broadly distributed along the PM surface. It is possible that the lack of organization of Ca_V_1.2 channels along ER junctions in tsA-201 cells reflects a lack of contact points or excessive distance between the ER and the PM. In ventricular myocytes, the membrane binding protein junctophilin-2 (JPH2) has been suggested to tether the jSR to the t-tubule membrane (Takeshima et al., 2000). Thus, we attempted to anchor the ER to the PM by co-transfecting tsA-201 cells with JPH2. However, even in the presence of JPH2, the Ca_V_1.2 channel cluster distribution was not limited to PM-ER junctions in the manner that they are in cardiomyocytes (Figure 3—figure supplement 1B). These data suggest that Ca_V_1.2 channel clustering occurs independently of SR/ER microdomains.

To determine the number of channels within Ca_V_1.2 clusters, we injected mice with an adeno-associated virus serotype 9 (AAV9) designed to express photo-activatable-GFP–tagged Ca_v_β_2a_, and examined ventricular myocyte Ca_V_1.2 clusters 5 wk later using single-particle photobleaching (Ulbrich and Isacoff, 2007). Ca_V_β_2a_ is a palmitoylated peripheral membrane protein that binds to the α_1_ pore-forming subunit of Ca_V_1.2 with a 1:1 stoichiometry (Dalton et al., 2005); therefore, photo-activation of this protein with 405-nm light provides a fluorescent marker of Ca_V_1.2 channels. Single Ca_V_1.2 were identified and excited using total internal reflection fluorescence (TIRF) microscopy. The number of channels in each cluster was determined by continuous photobleaching and counting of stepwise decreases in fluorescence intensity (Figure 2D–F). A preponderance (47%) of Ca_V_1.2 clusters displayed 1 to 6 stepwise decreases in fluorescence (Figure 2F). A single photobleaching step was observed in only 1% of the spots analyzed. Indeed, the mean number of bleaching steps per cluster was 7.91 ± 0.23 (*n* = 25). This suggests that Ca_V_1.2 channels preferentially cluster in groups of about 8 channels in adult ventricular myocytes. We did not discriminate cluster size based on location; thus, our estimate of 8 channels/cluster combines dyadic and extra-dyadic populations.

Similar stepwise photobleaching experiments were performed on enhanced green fluorescent protein (EGFP)-tagged Ca_V_1.2 channels heterologously expressed in tsA-201 cells (Figure 3D–F). TIRF imaging showed that Ca_V_1.2 clusters displayed a mean of 5.07 ± 0.15 (*n* = 10) discrete bleaching steps. Taken together with the data from ventricular myocytes, these findings suggest that the formation of multi-channel clusters is a fundamental property of Ca_V_1.2 channels with important implications for Ca^2+^ signaling.

### Spontaneous Ca_V_1.2 channel coupling occurs via Ca^2+^-dependent physical interactions between adjacent channel C-termini

**Figure 4.**
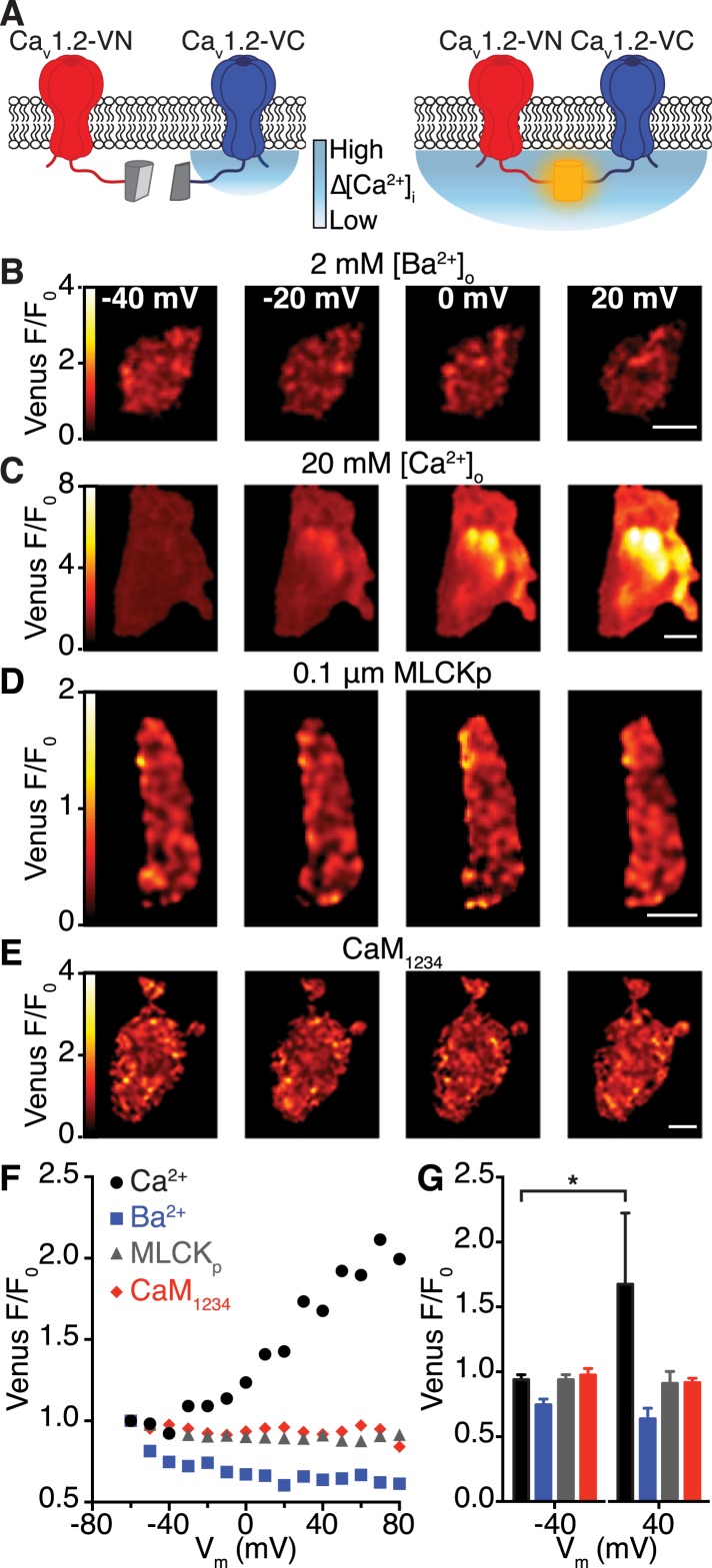
Interactions between Ca_V_1.2 channel C-termini occur spontaneously and in a Ca^2+^/CaM-dependent manner. (**A**) Illustration of the bimolecular fluorescence complementation strategy for assaying interactions between Ca_V_1.2 channel C-termini. Non-interacting channels tagged at their C-terminus with either the N- or C-terminal half of split Venus are non-fluorescent (*left*). Spontaneous interactions between channel C-termini result in reconstitution of Venus and emission of fluorescence (*right*). (**B**–**E**) TIRF images obtained from whole-cell patch-clamped tsA-201 cells expressing Ca_V_1.2-VN and Ca_V_1.2-VC over 9-s voltage steps to the indicated potentials. Images were median-filtered, smoothed, pseudo-colored with a ‘red-hot’ LUT, and divided by the initial −60 mV image to obtain calibrated Venus F/F_0_. Experiments were performed with 2 mM Ba^2+^ (**B**) or 20 mM Ca^2+^ (**C**–**E**) in the perfusing solution. Scale bars = 3 μm. (See also Figure 4—figure supplement 2) (**D**) Images obtained during dialysis with MLCKp (0.1 μM). (**E**) Images from a cell in which CaM_1234_ was co-expressed with Ca_V_1.2-VN and Ca_V_1.2-VC (see also Figure 4—figure supplement 3). (**F**) Relationship between membrane voltage and Venus reconstitution for each experimental condition. (**G**) Bar chart showing mean Venus fluorescence (F/F_0_) ± SEM for each condition at −40 and +40 mV (*p < 0.05). **DOI:** http://dx.doi.org/10.7554/eLife.05608.008

To investigate the mechanisms regulating Ca_V_1.2-Ca_V_1.2 interactions in living cells (Figure 4), we applied a bimolecular fluorescence complementation approach using Ca_V_1.2 channels fused with either the N- or C-terminus of the split-Venus fluorescent protein system to yield Ca_V_1.2-VN155(I152L) and Ca_V_1.2-VC155, respectively. In isolation, VN155(I152L) and VC155 are non-fluorescent; however, when brought into close proximity by interacting proteins, they can reconstitute a full, fluorescent Venus protein. Thus, Venus fluorescence can be used to report spontaneous interactions between adjacent Ca_V_1.2 channels, as depicted in Figure 4A. In cells expressing Ca_V_1.2-VN155(I152L) and Ca_V_1.2-VC155 channels, Venus fluorescence at −80 mV was very low, suggesting that Ca_V_1.2-Ca_V_1.2 channel interactions are rare at this hyperpolarized membrane potential. To determine whether an increase in [Ca^2+^]_i_ is *sufficient* to induce Ca_V_1.2-Ca_V_1.2 channel interactions, we loaded tsA-201 cells with DMNP-EDTA (caged Ca^2+^) via the patch pipette and induced flash photolysis of DMNP-EDTA with pulses of 405-nm light while holding cells at −80 mV. Photolysis of DMNP-EDTA induced a transient increase in [Ca^2+^]_i_ and a concomitant increase in Venus fluorescence (Figure 4—figure supplement 1), demonstrating that an elevation in [Ca^2+^]_i_ is indeed *sufficient* to induce Ca_V_1.2-Ca_V_1.2 interactions.

**Figure 4—figure supplement 1.**
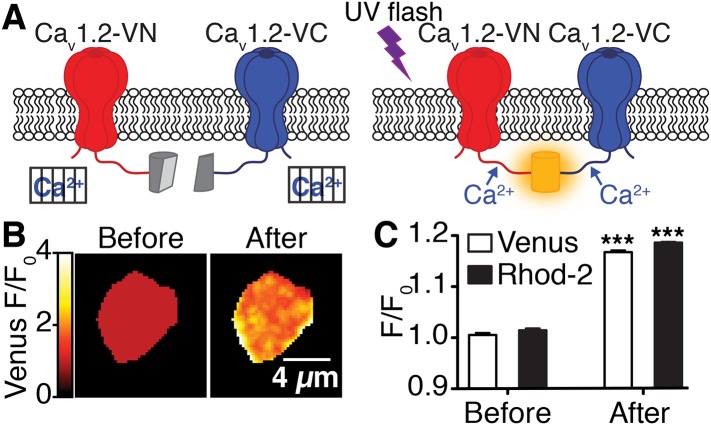
Flash photolysis of caged Ca^2+^ stimulates Ca_V_1.2 interactions. (**A**) Illustration of flash photolysis-induced bimolecular fluorescence complementation. Prior to uncaging of Ca^2+^, cells were bathed in a zero Ca^2+^ solution; under these conditions, split-Venus–tagged channels do not interact (*left*). Upon application of a UV flash to uncage Ca^2+^, interactions between the channel C-termini results in reconstitution of Venus protein and emission of fluorescence (*right*). (**B**) Confocal images of Venus fluorescence emission (F/F_0_) before and after flash photolysis of caged Ca^2+^. (**C**) The bar chart shows mean Venus and Rhod-2 fluorescence emission (F/F_0_) ± SEM before and after Ca^2+^ uncaging (***p < 0.001, for comparison of Ca_V_1.2interactions [Venus reconstitution] and intracellular Ca^2+^ concentration [Rhod-2 emission] before and after flash photolysis of caged Ca^2+^; paired *t*-test). **DOI:** http://dx.doi.org/10.7554/eLife.05608.009

**Figure 4—figure supplement 2.**
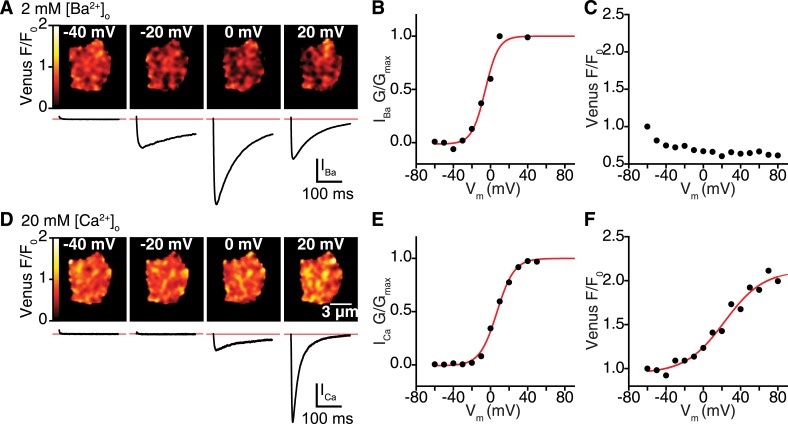
Ca_V_1.2 interactions are Ca^2+^ dependent. (**A**–**C**) Venus fluorescence and Ca_V_1.2 currents were recorded in whole-cell mode from tsA-201 cells expressing Ca_V_1.2-VN and Ca_v_1.2-VC during depolarizing voltage steps to −40, −20, 0 and +20 mV with Ba^2+^ as the charge carrier (2 mM [Ba^2+^]_o_). Voltage dependencies were fit with a Boltzmann sigmoidal function (red solid line) except in (**C**), where Venus F/F_0_ decayed exponentially during the voltage protocol in a manner reminiscent of photobleaching. (**A**) Calibrated Venus F/F_0_ TIRF images (*top*) and I_Ba_ (*bottom*) from a representative tsA-201 cell. (**B**) Voltage dependence of normalized conductance. (**C**) Venus fluorescence (F/F_0_) plots. (**D**–**F**) Venus fluorescence and Ca_V_1.2 currents were recorded as above (**A**–**C**) with Ca^2+^ as the charge carrier (20 mM [Ca^2+^]_o_). (**D**) Calibrated Venus F/F_0_ TIRF images (*top*) and I_Ba_ (*bottom*) from a representative tsA-201 cell. (**E**) Voltage dependence of normalized conductance. (**F**) Venus fluorescence (F/F_0_) plots. Voltage dependencies were fit with a Boltzmann sigmoidal function (red solid line). **DOI:** http://dx.doi.org/10.7554/eLife.05608.010

**Figure 4—figure supplement 3.**
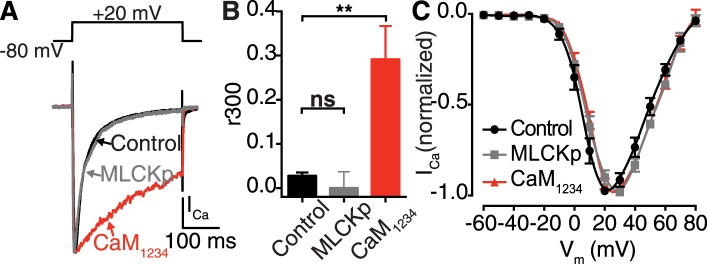
Ca^2+^ binding to distinct CaM pools regulates CDI. (**A**–**C**) tsA-201 cells expressing Ca_V_1.2 were subjected to a 300-ms depolarization from a holding potential of −80 mV to a test potential of +20 mV in the presence of 20 mM [Ca^2+^]_o_. (**A**) I_Ca_ elicited under control conditions (20 mM [Ca^2+^]_o_), with 0.1 μM MLCKp dialyzed via the patch pipette, and in cells co-expressing Ca^2+^-insensitive CaM_1234_. (**B**) Mean r_300_ ratios ± SEM (**p = 0.002). (**C**) Current-voltage relationships. **DOI:** http://dx.doi.org/10.7554/eLife.05608.011

**Figure 4—figure supplement 4.**
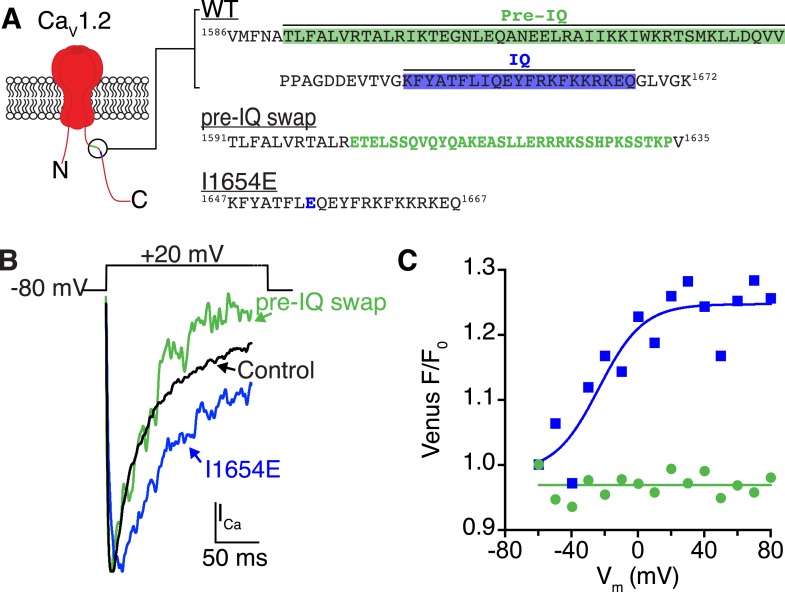
Ca^2+^/CaM binding to the pre-IQ domain, and not the IQ domain, mediates channel coupling. (**A**) Illustration of the pore-forming subunit of a Ca_V_1.2 channel embedded in the PM, with intracellular N and C-termini. The C-terminus contains pre-IQ (*green*) and IQ (*blue*) motifs, which are known to bind CaM. The amino acid sequence of the pre-IQ and IQ segments of the C-terminus of WT, pre-IQ swap, and I1654E mutant channels used in this study are shown on the *right*. For the pre-IQ swap, a 33-amino-acid segment was replaced with 33 non-identical amino acids. For the I1654E mutation, a point mutation was made replacing I1654 with E. (**B**) I_Ca_ elicited by a depolarizing step from −80 mV to a test potential of +20 mV under control conditions (20 mM [Ca^2+^]_o_) in tsA-201 cells expressing WT (*black*), I1624E (*blue*), or pre-IQ swap (*green*) Ca_V_1.2 channels. (**C**) Venus fluorescence (F/F_0_) plots for the two mutant channels. Ca_V_1.2(I1624E) channels exhibited a voltage-dependent increase in Venus reconstitution that fit a Boltzmann sigmoidal function (blue solid line; *n* = 5). Venus F/F_0_ decayed over the course of the voltage protocol in cells expressing Ca_V_1.2(pre-IQ swap) (*n* = 8). **DOI:** http://dx.doi.org/10.7554/eLife.05608.012

**Figure 4—figure supplement 5.**
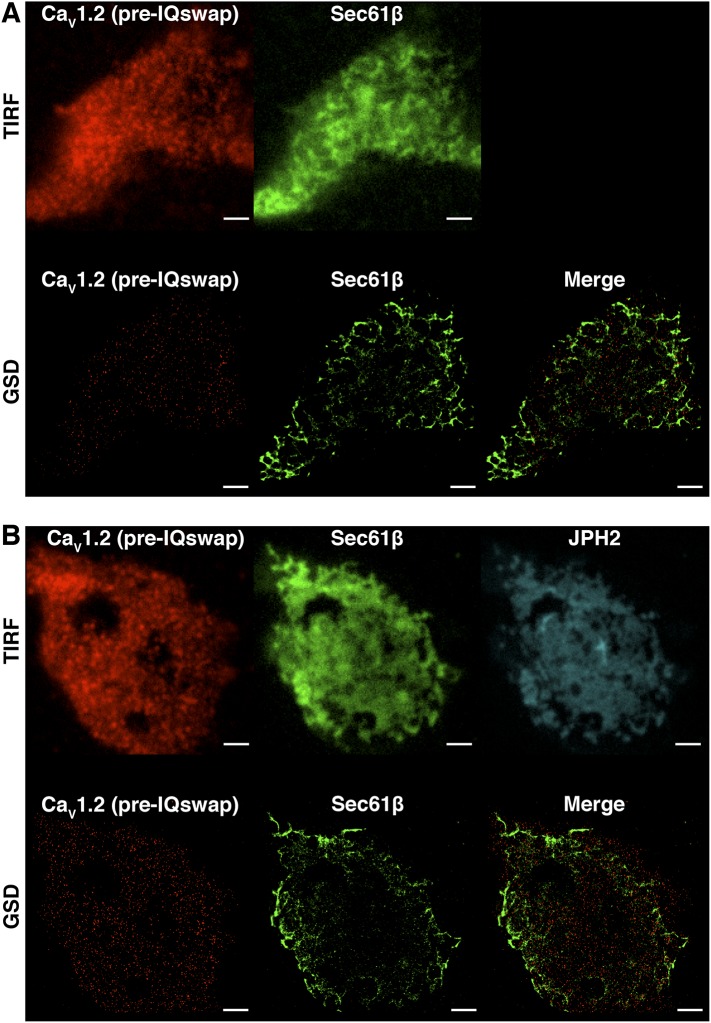
Ca_V_1.2 channel clustering is necessary but not sufficient for functional coupling. (**A**) TIRF (*top*) and super-resolution GSD (*bottom*) images of immunolabeled Ca_V_1.2(pre-IQ swap) channels (*left*) and mCherry-Sec61β (*middle*) in an exemplary transfected tsA-201 cell. The image on the bottom right was generated by merging Ca_V_1.2(pre-IQ swap) and mCherry-Sec61β GSD images. (**B**) *Top*: TIRF images of immunolabeled Ca_V_1.2(pre-IQ swap) channels (*left*), mCherry-Sec61β (*middle*), and JPH2-GFP (*right*) in a transfected tsA-201 cell. *Bottom*: GSD images from the same cell showing Ca_V_1.2(pre-IQ swap) channels (*left*), mCherry-Sec61β (*middle*), and a merge of the two (*right*). Scale bars = 2 μm. **DOI:** http://dx.doi.org/10.7554/eLife.05608.013

To determine if Ca^2+^ influx via Ca_V_1.2 channels is *required* for channel interactions, we depolarized cells and recorded Venus fluorescence and membrane currents in the presence of Ba^2+^ or Ca^2+^. Cells were dialyzed with an intracellular solution containing 10 mM EGTA to restrict the local [Ca^2+^]_i_ signal to about 1 μm from the channel and maintain very low global [Ca^2+^]_i_. With Ba^2+^ in the external solution, depolarization evoked currents (I_Ba_) over a wide range of potentials, but Venus fluorescence was very low at all membrane potentials (Figure 4B,F,G and Figure 4—figure supplement 2A–C). After switching to a Ca^2+^-containing external solution, application of the same voltage protocol activated currents (I_Ca_) and induced graded increases in Venus fluorescence (Figure 4C,F,G and Figure 4—figure supplement 2D–F). The fluorescence-voltage and I_Ca_ conductance-voltage relationships were sigmoidal. The normalized conductance and Venus fluorescence exhibited similar voltage dependencies (Figure 4—figure supplement 2E,F). Taken together with super-resolution, photobleaching and DMNP-EDTA data, these findings suggest that local and global [Ca^2+^]_i_ signals produced by Ca^2+^ influx via Ca_V_1.2 channels are *required* for physical interactions between adjacent channels within a cluster.

### Ca^2+^/CaM binding to the pre-IQ domain mediates functional coupling, but not clustering, of Ca_V_1.2 channels

We next investigated the mechanisms underlying Ca^2+^-dependent coupling of Ca_V_1.2 channels, focusing on CaM since this protein binds Ca^2+^, associates with Ca_V_1.2 C-terminal pre-IQ and IQ domains, and is involved in CDI and CDF of Ca_V_1.2 channels (Eckert and Tillotson, 1981). Dialysis with an intracellular solution containing the CaM inhibitory peptide MLCKp (0.1 μM) prevented Venus reconstitution during membrane depolarization (Figure 4D,F,G), without altering the rate of inactivation of I_Ca_ (Figure 4—figure supplement 3). Expression of a mutant CaM that does not bind Ca^2+^ (CaM_1234_) also prevented Ca_V_1.2-VN-Ca_V_1.2-VC fusion upon membrane depolarization (Figure 4E,F,G). However, unlike MLCKp, CaM_1234_ did slow the rate of I_Ca_ inactivation (Figure 4—figure supplement 3), suggesting that two functionally distinct CaM molecules are involved in CDI and Ca_V_1.2-to-Ca_V_1.2 channel interactions.

Given the apparently essential role of Ca^2+^/CaM, we next examined the importance of IQ and pre-IQ motif CaM-binding sites for Ca_V_1.2-Ca_V_1.2 channel interactions. To do so, we mutated critical residues in each segment and used bimolecular fluorescence complementation to evaluate changes in channel coupling ability. Ca_V_1.2-VN and Ca_V_1.2-VC channels with an I1654E mutation in their IQ domains were able to fuse upon membrane depolarization (Figure 4—figure supplement 4). This isoleucine residue is crucial for CaM binding to the IQ motif. Previous in vitro studies have reported that the I1654E (or the human homolog I1624E) mutation decreases the affinity of the IQ motif for CaM by ∼100-fold (Zühlke et al., 1999, 2000). Thus, our results suggest that CaM binding to the IQ-motif is not required for Ca_V_1.2 channel coupling. To investigate the role of the pre-IQ motif in channel interactions, we exchanged a 33-amino-acid segment of the pre-IQ domain for 33 non-identical amino acids (Figure 4—figure supplement 4A). A similar segment exchange performed on human Ca_V_1.2 channels expressed in tsA-201 cells was previously shown to impair channel clustering on the PM and reduce P_o_ compared to wild-type (WT) channels (Kepplinger et al., 2000). Interestingly, the pre-IQ ‘swap’ mutation rendered Ca_V_1.2-VN and Ca_V_1.2-VC channels incapable of functional coupling (Figure 4—figure supplement 4C). Collectively, these results suggest that pre-IQ domains are required for Ca^2+^/CaM-dependent Ca_V_1.2 channel oligomerization. Additional credence is given to this finding by the previously published crystal structure of dimeric cardiac L-type Ca^2+^ channels showing two pre-IQ helices bridged by two Ca^2+^/CaMs (Fallon et al., 2009).

We next examined the spatial distribution of Ca_V_1.2(pre-IQ swap) channels in tsA-201 cells co-transfected with mCherry-sec61β (to permit visualization of the ER). Surprisingly, Ca_V_1.2(pre-IQ swap) channels still formed clusters in tsA-201 cells despite their inability to functionally interact (Figure 4—figure supplement 5A). Indeed, under identical imaging conditions (i.e., using the same fixative and TIRF penetration depth), Ca_V_1.2(pre-IQ swap) channel cluster areas were not significantly different from those of WT channels. As noted above for WT channels, the Ca_V_1.2(pre-IQ swap) channel cluster distribution was not limited to PM-ER junctions, even in the presence of JPH2 (Figure 4—figure supplement 5A,B). These results suggest that, while the physical proximity of Ca_v_1.2 channels is necessary for channel interactions, it is not sufficient for functional coupling of the channels.

### Ca_V_1.2 channel coupling augments activity

**Figure 5.**
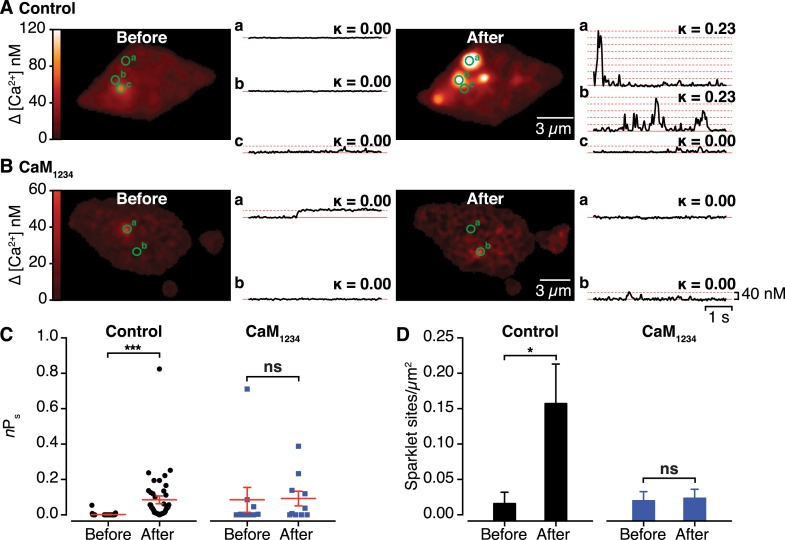
Effects of interactions between Ca_V_1.2 channel C-termini on channel activity. (**A** and **B**) Calibrated TIRF images (see also Video 2) of representative control tsA-201 cells (**A**) and tsA-201 cells expressing the Ca^2+^-insensitive CaM_1234_ mutant (**B**). In both cases, cells expressed Ca_V_1.2-VN and Ca_V_1.2-VC and were loaded with Rhod-2 via the patch pipette. Cells were held at −80 mV during sparklet recordings before (*left*) and after (*right*) depolarization to +60 mV. The time course of [Ca^2+^]_i_ for each sparklet site (denoted by green circles) before and after depolarization is shown to the right of each image. (**C** and **D**) Scatter plots of sparklet activity (**C**; *n*P_s_; ***p *<* 0.001) and sparklet site density (**D**; *p = 0.03), before and after depolarization in control (*n* = 5) and CaM_1234_-expressing (*n* = 6) cells. ns, not significant. **DOI:** http://dx.doi.org/10.7554/eLife.05608.014

An important prediction of our findings is that the functional coupling produced by Ca^2+^-induced Ca_V_1.2-Ca_V_1.2 channel interactions manifests as enhanced channel activity. To test this, we exploited the fact that Venus reconstitution is irreversible, recording Ca_V_1.2 sparklets (Wang et al., 2001; Navedo et al., 2005) before and after Ca^2+^-induced Ca_V_1.2 coupling during membrane depolarization. Prior to membrane depolarization, Ca_V_1.2 sparklet activity (*n*P_s_) was low (0.002 ± 0.001). However, after depolarization, *n*P_s_ increased ∼40-fold (0.085 ± 0.022) and Ca_V_1.2 sparklet density increased almost 10-fold (Figure 5A,C,D and Video 2; *n* = 5 cells). Membrane depolarization failed to significantly alter *n*P_s_ or Ca_V_1.2 sparklet density in cells co-expressing the Ca^2+^-insensitive CaM_1234_ mutant (Figure 5B–D; *n* = 6 cells). These data indicate that the post-depolarization augmentation of Ca_V_1.2 sparklet activity resulted from a Ca^2+^/CaM-dependent increase in the activity of previously active sites as well as the emergence of new, high-activity Ca^2+^ sparklet sites that appear to lack CDI. These findings suggest that Ca_V_1.2 channel interactions increase Ca^2+^ influx and, consequently, total conductance by increasing the extent to which the channels are functionally coupled.

**Video 2.** Ca^2+^ sparklet activity and site density are augmented by Ca_V_1.2 channel interactions. Stacks of 2D images acquired at a frame rate of 100 Hz from a representative, Rhod-2–dialyzed tsA cell expressing Ca_V_1.2-VN and Ca_V_1.2-VC, held at −80 mV, before (*left*) and after (*right*) depolarization. **DOI:** http://dx.doi.org/10.7554/eLife.05608.015

### Ca_V_1.2 channel coupling is dynamic and transiently persistent

**Figure 6.**
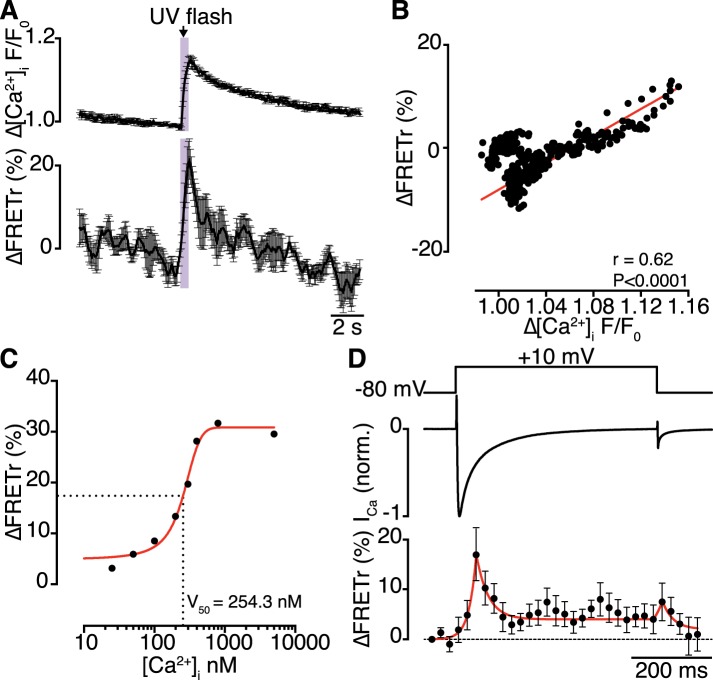
Interactions of Ca_V_1.2 C-termini occur dynamically with Ca^2+^ influx and are transiently persistent. (**A**) Time course of the percent change in FRETr (*bottom*) evoked by flash photolysis of caged Ca^2+^ (purple box). Experiments were performed at a holding potential of −80 mV with zero EGTA or BAPTA; thus, the change in [Ca^2+^]_i_ (F/F_0_; *top*) was global. Averaged traces (*n* = 5 cells) and error bars showing SEM at each sampling point. (**B**) Correlation between changes in FRETr and global [Ca^2+^]_i_ produced by caged Ca^2+^ photolysis, showing that increases in [Ca^2+^]_i_ were accompanied by an increase in FRETr (p < 0.0001). (**C**) Plot of the percent change in FRETr vs [Ca^2+^]_i_ (nM). Data (*n* = 8 cells) were fit to a Boltzmann Sigmoidal function (solid red line) with V_50_ = 254.3 nM (black dashed line). (**D**) Step depolarization (*top*) in the presence of 10 mM EGTA produced local [Ca^2+^]_i_ elevation and inactivating I_Ca_ (*middle*). Increased channel interactions, represented as the percent change in FRETr (*bottom;* averaged from *n* = 6 cells), were detected at the onset of depolarization and repolarization during the peak and tail currents. Dashed line shows FRETr baseline level. **DOI:** http://dx.doi.org/10.7554/eLife.05608.016

Having demonstrated that physical Ca_V_1.2-to-Ca_V_1.2 interactions functionally couple adjoining channels to enhance channel activity, we investigated the dynamics of these interactions. Bimolecular fluorescence complementation experiments are unable to provide information about channel-interaction dynamics, since Venus reconstitution is irreversible (Kerppola, 2006). Instead, we sought to detect fluorescence resonance energy transfer (FRET) between EGFP and red fluorescent protein (RFP)-tagged Ca_V_1.2 channels as a function of [Ca^2+^]_i_. Flash photolysis of DMNP-EDTA (at −80 mV) induced a transient increase in [Ca^2+^]_i_ and enhanced the Ca_V_1.2-RFP/Ca_V_1.2-EGFP FRET ratio (FRETr; Figure 6A). The time courses of [Ca^2+^]_i_ and FRETr were similar (Figure 6B), with a time-to-peak of 605.8 ± 98.8 ms for [Ca^2+^]_i_ and 531.5 ± 57.5 ms for FRETr. The [Ca^2+^]_i_-FRETr relationship was sigmoidal, yielding a FRETr_1/2_ at a [Ca^2+^]_i_ of approximately 250 nM (Figure 6C). Both [Ca^2+^]_i_ and FRETr traces followed triple exponential decay kinetics; for [Ca^2+^]_i_, the decay time constants (τ) for fast, intermediate and slow components were 0.62 ± 0.15, 2.19 ± 0.40 and 3.15 ± 0.26 s, respectively, and the corresponding values for FRETr were 0.64 ± 0.48, 1.09 ± 0.97 and 3.71 ± 0.73 s (*n* = 5). The time to decay to 50% of the peak (T_50%_) was 2.65 ± 0.22 s for [Ca^2+^]_i_ and 0.63 ± 0.23 s for FRETr. The complex multi-component decay kinetics of each trace reflects the multiple Ca^2+^ binding sites and affinities of the two lobes of CaM for Ca^2+^ (Faas et al., 2011; Faas and Mody, 2012).

Concurrent recordings of currents and FRETr during membrane depolarization in the presence of Ca^2+^ (with 10 mM EGTA in the pipette solution) showed that depolarization to +10 mV evoked a transient increase in FRETr (Figure 6D). The increase in FRETr appeared biphasic, with a large amplitude spike followed by a lower level plateau that was sustained for the duration of the increase in [Ca^2+^]_i_. These data suggest that Ca_V_1.2-Ca_V_1.2 interactions are dynamic and regulated by local and global changes in [Ca^2+^]_i_. Notably, the increase in FRETr persisted after the Ca^2+^ current had decayed to baseline, indicating that channels remained coupled for a time in the absence of a stimulus.

### Ca_V_1.2 channel coupling facilitates I_Ca_

An explicit implication of our results is that physical Ca_V_1.2-Ca_V_1.2 interactions are critical for CDF in ventricular myocytes. To test this hypothesis, we investigated whether Ba^2+^ permeation and inhibition of CaM with MLCKp—both of which prevent Ca_V_1.2-Ca_V_1.2 interaction (see above)—decreases or eliminates CDF in ventricular myocytes. Because Ca^2+^/CaM-dependent kinase II (CaMKII) has been implicated in Ca^2+^ current facilitation (Anderson et al., 1994; Xiao et al., 1994; Yuan and Bers, 1994), we included 10 mM EGTA in the patch pipette to maintain global [Ca^2+^]_i_ < 50 nM, which is below the threshold (>200 nM) for activation of this kinase (Miller and Kennedy, 1986). Experiments were performed in cells dialyzed with 100 nM or 1 μM MLCKp. The rationale for using these two concentrations of MLCKp is that, whereas 100 nM MLCKp inhibits CaM (Török and Trentham, 1994; Török et al., 1998), we found that it does not change CaMKII activity (p < 0.05). However, we found that 1 μM MLCKp inhibits CaM and eliminates CaMKII activity. Thus, using these two MLCKp concentrations, we can selectively dissect the contribution of CaM and locally activated (i.e., near the channel pore) CaMKII.

**Figure 7.**
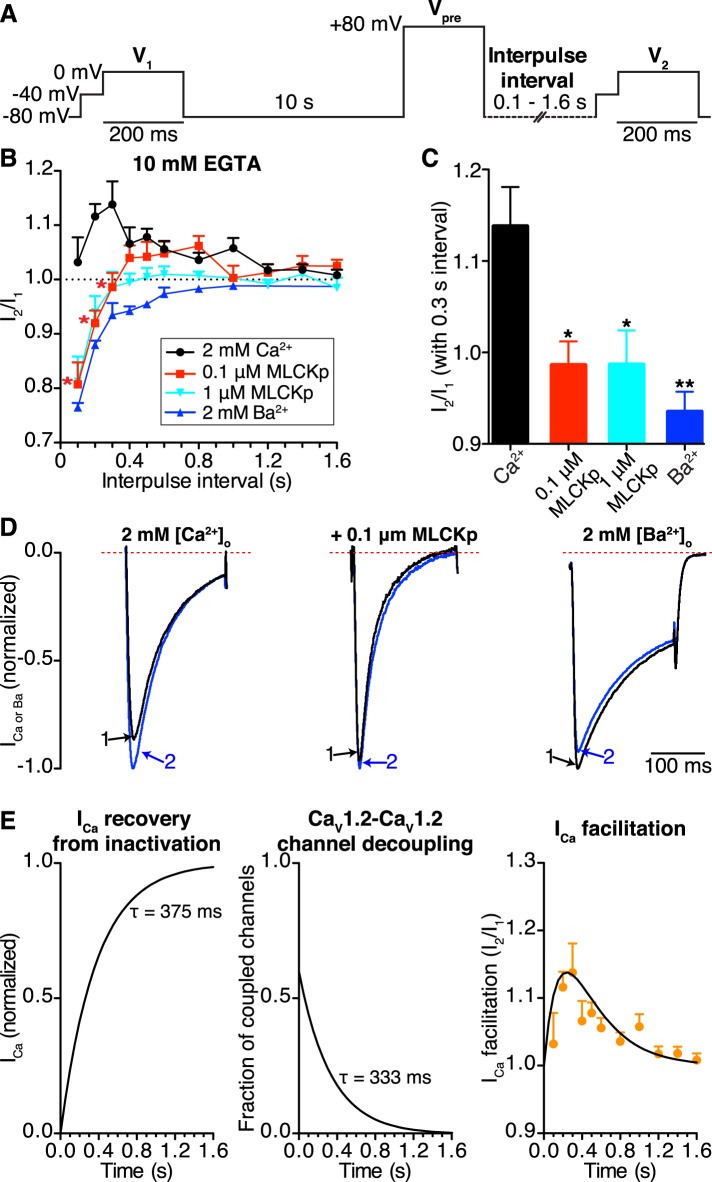
Ca_V_1.2-to-Ca_V_1.2 channel coupling is critical for I_Ca_ facilitation in cardiomyocytes. (**A**) Voltage protocol used to evoke I_1_ and I_2_ in ventricular myocytes. (**B**) Line chart summarizing the current amplitude ratio (I_2_/I_1_) at 0 mV for each condition over the range of interpulse intervals from 0.1 to 1.6 s. (**C**) Bar chart summarizing the current-amplitude ratio (I_2_/I_1_) with a fixed interpulse interval of 300 ms for each condition. (**D**) Normalized whole-cell currents evoked by the protocol in (**A**), with 2 mM [Ca^2+^]_o_ as the charge carrier without (*left*) or with (*middle*) intracellular dialysis of 0.1 μM MLCKp (*middle*). The currents on the right were recorded with 2 mM [Ba^2+^]_o_ as the charge carrier. Data are shown as means + SEM (*p < 0.05, **p < 0.01 vs control (2 mM [Ca^2+^]_o_)). (**E**) Simulated time-course of I_Ca_ recovery from inactivation (*left*), Ca_V_1.2-Ca_V_1.2 channel cluster disassembly (*middle*), and I_Ca_ facilitation (solid black line; *right*). Recovery and channel decoupling curves are single exponential functions with time constants (τ) of 375 ms and 333 ms, respectively. The I_Ca_ facilitation curve is the product of the recovery and decoupling functions. For comparison, the experimental facilitation data (orange circles) collected with 2 mM [Ca^2+^]_o_ as the charge carrier is plotted alongside the simulated I_Ca_ facilitation data. The amplitude of the decoupling function was scaled to fit the facilitation data. **DOI:** http://dx.doi.org/10.7554/eLife.05608.017

We used a three-step protocol (Figure 7A) to record facilitated Ca_V_1.2 currents as previously described (Poomvanicha et al., 2011). Briefly, cells were held at −80 mV and I_Ca_ was elicited with a 200-ms control pulse (V_1_) to 0 mV. Cells were held again at −80 mV for 10 s followed by a 200-ms prepulse to +80 mV (V_pre_). In light of our FRET results suggesting that Ca_V_1.2 channels remain coupled for a time in the absence of a stimulus, we held the cells at −80 mV for a variable interpulse interval of 0.1–1.6 s before beginning the second 200-ms test pulse (V_2_) to 0 mV. Fast Na^+^ currents were inactivated with a 50-ms step to −40 mV applied prior to each control (V_1_) or test pulse (V_2_). The ratio of I_Ca_ resulting from test and control pulses (I_2_/I_1_) was used as a measure of facilitation (I_2_/I_1_ > 1) and recovery from inactivation (I_2_/I_1_ ≈ 1).

In control cells (no peptide, 2 mM external Ca^2+^), this protocol induced I_Ca_ facilitation (i.e., I_2_/I_1_ > 1) (Figure 7B). Note, however, that the magnitude of I_Ca_ facilitation varied with interpulse duration, reaching a peak at a V_pre_-V_2_ interval of 0.3 s. Longer V_pre_-V_2_ intervals induced progressively less I_Ca_ facilitation. Indeed, I_2_/I_1_ was statistically indistinguishable from 1 at interpulse durations longer than 1.2 s, suggesting no I_Ca_ facilitation at stimulation rates >0.8 Hz.

In the presence of extracellular Ca^2+^, intracellular dialysis with 0.1 μM MLCKp suppressed I_Ca_ facilitation at interpulse intervals of 0.1–0.3 s (*n* = 10 for 0.1 s intervals; *n* = 5 for 0.2 and 0.3 s intervals), but control levels of facilitation returned with intervals of 0.4–1 s (*n* = 5–9, p < 0.05; Figure 7B). Dialysis with a 10-fold higher concentration of MLCKp (1 μM) eliminated I_Ca_ facilitation at all interpulse intervals (*n* = 4; Figure 7B). Finally, superfusion of ventricular myocytes with Ba^2+^ was as effective as dialysis with 1 μM MLCKp in preventing I_Ca_ facilitation (Figure 7B–D).

We performed a detailed analysis of our data to gain more insights into the relationship between Ca_V_1.2 channel coupling and I_Ca_ facilitation during high frequency stimulation. The amplitude of I_2_ in the paired pulse facilitation would depend, in part, on the degree of recovery from inactivation. A previous study has determined that the time course of I_Ca_ recovery from inactivation follows a single exponential function with a τ_recovery_ of about 375 ms (Blaich et al., 2010). Our FRET data in Figure 6D suggest that the number of coupled Ca_V_1.2 channels will fade exponentially with an estimated τ_decoupling_ of 333 ms upon repolarization. In Figure 7E (*left*), we show a simulation of the time-course of I_Ca_ recovery and Ca_V_1.2 channel decoupling (*middle*) during repolarization using exponential functions with these τ_decoupling_ and τ_recovery_ values. Note that the time-course of I_Ca_ facilitation data obtained from myocytes superfused with Ca^2+^ is well described by the product of the recovery and decoupling functions (Figure 7E; *right*). This analysis is consistent with a model in which I_Ca_ facilitation during high frequency stimulation is directly proportional to the number of coupled channels and the number of channels available for activation.

These data support the view that I_Ca_ facilitation in ventricular myocytes depends on Ca^2+^ influx and CaM, but has CaMKII-dependent and independent components. In combination with our FRET and split-Venus data, these findings suggest that Ca^2+^ augments Ca_V_1.2 channel activity at least in part by increasing the number of functionally coupled channels.

## Discussion

**Figure 8.**
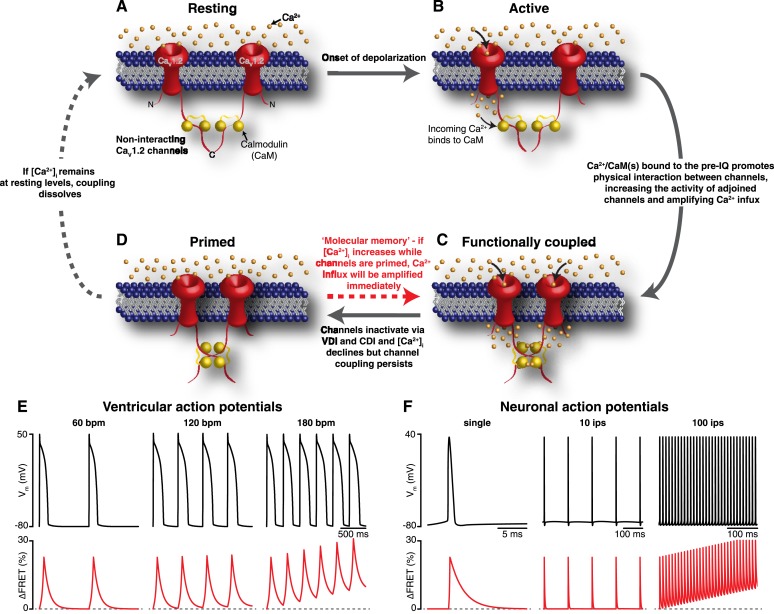
Mechanism and proposed model for the functional coupling of Ca_V_1.2 channels. (**A**) Ca_V_1.2 channels are arranged into clusters in the PM of excitable cells; for simplicity, a cluster of two channels is shown. At the resting membrane potential (e.g., −80 mV), [Ca^2+^]_i_ and Ca_V_1.2 *P*_o_ are low; hence, the majority of Ca_V_1.2 channels are non-interacting. (**B**) During an action potential, the PM becomes depolarized, increasing the *P*_o_ of independently gating Ca_V_1.2 channels. Ca^2+^ flows into the cell through these active channels, producing an elevation in local [Ca^2+^]_i_ and increasing Ca^2+^ binding to CaM. (**C**) Ca^2+^/CaM binding to the C-terminal pre-IQ domain of the Ca_V_1.2 channel promotes physical interactions between adjacent channels. This functional coupling increases the activity of adjoined channels and thus amplifies Ca^2+^ influx. (**D**) Ca_V_1.2 channels undergo VDI and CDI, and [Ca^2+^]_i_ declines once more. However, the channels remain coupled in a ‘primed’, non-conducting state for a finite time. If the membrane is depolarized again when the channels are still primed, the amplification of Ca^2+^ influx will be immediate; otherwise, if [Ca^2+^]_i_ remains at resting levels beyond the lifetime of the primed state, the coupling dissolves and the cycle begins again. (**E**) and (**F**) show proposed rate-dependent changes in Ca_V_1.2 channel coupling in ventricular myocytes and neurons, respectively. *Top*: Simulated ventricular and neuronal action potentials are depicted at low, intermediate, and high firing rates. *Bottom*: The accompanying dynamic change in Ca_V_1.2 channel coupling (reflected by FRET changes between adjacent channels). bpm, beats per minute; ips, impulses per second. **DOI:** http://dx.doi.org/10.7554/eLife.05608.018

Our results support a new model for Ca_V_1.2 channel function. An illustration of our proposed model for coupled gating of Ca_V_1.2 channels is shown in Figure 8A–D. In our formulation, CDI and CDF are interrelated processes, both dependent on the coupling state of adjacent Ca_V_1.2 channels. The C-terminal tail of the channel serves a dual role: inducing the functional coupling of adjacent channels via protein-to-protein interactions and regulating channel open probability. The physical interaction between clustered Ca_V_1.2 channels is tightly regulated by local and global [Ca^2+^]_i_. The cascade of events that culminates in the coupling of Ca_V_1.2 channels during an action potential begins with the gating of an individual channel within a cluster. The resulting Ca_V_1.2 sparklet induces the binding of Ca^2+^ to CaM in the pre-IQ domain of the channel, which promotes physical interactions between contiguous channels. This increases the activity of adjoined channels, elevating local [Ca^2+^]_i_. As individual channels within a cluster undergo VDI and CDI and close, [Ca^2+^]_i_ decreases and coupled channels disassemble. This, in turn, decreases channel opening probability and terminates Ca^2+^ flux. Thus, the overall activity of Ca_V_1.2 channels within a cluster depends on the number of channels that form dimers or higher-order oligomers.

Our results have profound implications for current models of cardiac EC coupling as they provide an answer to a long-standing question in the field: What are the mechanisms that allow the simultaneous, coordinated opening of multiple Ca_V_1.2 channels near the jSR? EC coupling starts with membrane depolarization. According to our model, membrane depolarization increases the probability of Ca_V_1.2 sparklet occurrence. Ca_V_1.2 sparklets elevate local [Ca^2+^]_i_, thereby increasing the number of channels that form dimers or higher-order oligomers. This physical coupling increases the probability of synchronous, coincident openings of channels within a cluster (∼5–10 channels) to reliably activate nearby RyRs through Ca^2+^-induced Ca^2+^ release (Inoue and Bridge, 2003; Sobie and Ramay, 2009). Accordingly, the strength of cardiac contraction would depend, at least in part, on the number of physically and functionally coupled Ca_V_1.2 channels.

Our data further suggest that the degree of Ca_V_1.2-Ca_V_1.2 coupling varies within the physiological range of [Ca^2+^]_i_ reached in ventricular myocytes during a cardiac cycle. We found that Ca_V_1.2 channel coupling is dynamic and has an apparent K_d_ of ∼250 nM. While the model assumes that Ca_V_1.2-to-Ca_V_1.2 coupling is initiated by a Ca^2+^ sparklet, it could also be induced by a Ca^2+^ spark resulting from the opening of a small cluster of closely apposed RyRs. This could induce local Ca_V_1.2-to-Ca_V_1.2 coupling, even if transiently, priming these channels for opening during the action potential. Note, however, that because Ca^2+^ spark activity is very low during diastole, the number of coupled Ca_V_1.2 channels would likely be low. However, at the peak level of the [Ca^2+^]_i_ transient (∼700 nM) during EC coupling (Santana et al., 2002), the cell would have reached a maximal level of Ca_V_1.2-to-Ca_V_1.2 coupling.

An important finding in our study is that, while Ca_V_1.2 channel coupling fades as [Ca^2+^]_i_ decreases, it persists longer than the current that evoked it. This is important because Ca_V_1.2 channel activity remains elevated for as long as the channels remain coupled. Thus, by outlasting the [Ca^2+^]_i_ signal that evoked it, Ca_V_1.2 channel coupling acts as a type of ‘molecular memory’ that might serve to augment Ca^2+^ influx during repetitive membrane depolarization. As a consequence, an increase in action potential frequency can enhance Ca^2+^ influx via two mechanisms: first, it can increase [Ca^2+^]_i_, thereby increasing Ca_V_1.2 channel coupling; and second, if an AP arrives while a subpopulation of Ca_V_1.2 channels remain coupled, which could occur even if [Ca^2+^]_i_ had decreased to basal levels owing to the molecular memory phenomenon, it would encounter a cell with a higher number of coupled—that is, more active—channels (see Figure 8E,F). This molecular memory might also manifest itself as Ca_V_1.2 current facilitation (Marban and Tsien, 1982; Argibay et al., 1988; Gurney et al., 1989). In the case of cardiac muscle, Ca_V_1.2 current facilitation augments contractile force during increases in heart rate (Pieske et al., 1999). In neurons, facilitation of Ca_V_1.2 channels has been suggested to contribute to an intrinsic amplification of synaptic current and the enhancement of neuronal excitability (Powers and Binder, 2001; Striessnig et al., 2014).

Our data support the view that Ca^2+^ current facilitation in ventricular myocytes requires Ca^2+^ influx and CaM, but has CaMKII-dependent and -independent components. The latter likely involve Ca^2+^/CaM-induced Ca_V_1.2-Ca_V_1.2 channel coupling. Future experiments should investigate if CaMKII facilitates Ca^2+^ current in neurons and cardiac muscle by a similar mechanism.

Dynamic, [Ca^2+^]_i_-dependent Ca_V_1.2 channel coupling could have pathological repercussions. For example, coupling of a mutant channel with a higher intrinsic open probability to a WT channel could increase Ca^2+^ influx. One condition associated with aberrantly high Ca_V_1.2 channel gating is Timothy syndrome (TS), also known as long-QT syndrome 8. TS is an autosomal-dominant, multi-organ disorder caused by de novo gain-of-function missense mutations in exon 8 or an alternatively spliced exon 8A of Ca_v_1.2 (Splawski et al., 2004, 2005). Forced physical interactions between Ca_v_1.2 channels have an intriguing effect on adjoined channels: fusion of intrinsically hyperactive Ca_V_1.2-TS channels to WT channels induces these latter channels to function like TS channels. These findings have important implications. If TS channels can form stable interactions with neighboring WT channels in TS patients, then these mutant channels, which constitute only ∼23% of the total cardiac Ca_v_1.2 population, could have a disproportionally large effect on Ca^2+^ influx. We previously tested this idea in ventricular myocytes expressing our fusible Ca_v_1.2 channels and found that fusion of TS channels with WT channels led to the development of arrhythmogenic spontaneous SR Ca^2+^ release events in addition to increasing the amplitude of [Ca^2+^]_i_ transients and contractions (Dixon et al., 2012). These findings support the hypothesis that physical interactions between the C-termini of TS and WT channels produce a disproportionally large Ca^2+^ influx that ultimately induces arrhythmogenic changes in [Ca^2+^]_i_. Consistent with this, we found that the relationship between the level of Ca_V_1.2-TS channel expression and the probability of a Ca^2+^ wave is non-linear, suggesting that even low levels of these channels are sufficient to induce maximal changes in [Ca^2+^]_i_ (Drum et al., 2014).

Mutations in CaM have recently been linked to severe forms of long-QT syndrome, which are associated with life-threatening arrhythmias that occur very early in life (Limpitikul et al., 2014). Expression of these CaMs increases Ca_v_1.2 activity and open times, effects similar to those produced by the TS mutation. Because CaM regulates many other Ca^2+^ channel subtypes, including those that predominate in neurons, these mutant CaMs could lead to a multi-system disorder similar to TS. An important direction for future experiments will be to investigate whether Ca_v_1.2 channel dysfunction is associated with aberrant TSTS or TS-WT Ca_v_1.2 channel coupling or coupling of channels with mutant CaM to channels with WT CaM.

In summary, our study demonstrates that dynamic, Ca^2+^-driven physical interactions among clustered Ca_V_1.2 channels lead to cooperative gating of adjacent channels and enhanced Ca^2+^ influx. The physical proximity afforded by clustering of Ca_V_1.2 channels is necessary, but not sufficient, for functional coupling of the channels and occurs whether the channels are functionally coupled or not. Future studies should investigate the mechanisms that dictate the clustered arrangement of Ca_V_1.2 channels. It is likely that cooperative Ca_V_1.2 channel gating also plays an important role in physiological functions as diverse as neuronal excitability and rate-dependent increases in cardiac contraction, as well as pathological conditions such as long-QT syndrome.

## Materials and methods

### Isolation of ventricular myocytes

WT C57BL/6 mice were euthanized with a single lethal dose of sodium pentobarbital delivered via intraperitoneal injection, as approved by the University of Washington Institutional Animal Care and Use Committee (IACUC). Ventricular myocytes were isolated as previously described (Dixon et al., 2012). Briefly, the heart was excised and rinsed with cold 150 μM EGTA digestion buffer containing 130 mM NaCl, 5 mM KCl, 3 mM Na-pyruvate, 25 mM HEPES, 0.5 mM MgCl_2_, 0.33 mM NaH_2_PO_4_, and 22 mM glucose. The aorta was cannulated for Langendorff perfusion, and the coronary arteries were subsequently perfused with warmed (37°C) 150 μM EGTA digestion buffer until they were cleared of blood. The perfusate was then switched to digestion buffer (no EGTA) supplemented with 50 μM CaCl_2_, 0.04 mg/ml protease (XIV), and 1.4 mg/ml collagenase (type 2; Worthington Biochemical, Lakewood, NJ) for 8–10 min. The ventricles were then cut away from the atria, sliced, and placed in 37°C digestion buffer supplemented with 0.96 mg/ml collagenase, 0.04 mg/ml protease, 100 μM CaCl_2_, and 10 mg/ml bovine serum albumen (BSA). Gentle agitation was applied using a transfer pipette until the ventricles dissociated. The cells were then allowed to pellet by gravity for 15–20 min, after which they were washed in enzyme-free digestion buffer supplemented with 10 mg/ml BSA and 250 μM CaCl_2_, pelleted once more, and finally resuspended at room temperature in Tyrode's solution containing 140 mM NaCl, 5 mM KCl, 10 mM HEPES, 10 mM glucose, 2 mM CaCl_2_, and 1 mM MgCl_2_; pH was adjusted to 7.4 with NaOH.

### Plasmid constructs and tsA-201 cell transfection

tsA-201 cells (Sigma–Aldrich, St. Louis, MO) were cultured in Dulbecco's Modified Eagle Medium (DMEM; Gibco-Life Technologies, Grand Island, NY) supplemented with 10% fetal bovine serum (FBS) and 1% penicillin/streptomycin at 37°C in a humidified 5% CO_2_ atmosphere and passaged every 3–4 day. Cells were transiently transfected at ∼70% confluence using jetPEI (Polyplus Transfection, New York, NY) transfection reagent and plated onto the appropriate coverglass ∼12 hr before experiments. For super-resolution imaging and photobleaching experiments, cells were plated onto 22 mm no. 1.5 coverslips (Thermo Fisher Scientific, Waltham, MA). For all other experiments, cells were plated onto 25 mm no. 1 coverslips (Thermo Fisher Scientific). Plasmids used in this study include pcDNA clones of the pore-forming subunit of rabbit Ca_V_1.2 (α_1c_) and rat auxillary subunits Ca_V_α_2_δ and Ca_V_β_3_ (kindly provided by Dr Diane Lipscombe; Brown University, Providence, RI). Standard PCR techniques were used to fuse the carboxyl tail of Ca_V_1.2 to different proteins depending on the experimental approach: for FRET experiments, to EGFP or tagRFP; for bimolecular fluorescence complementation, to either the N-fragment (VN) or the C-fragment (VC) of the Venus protein (27097, 22011; Addgene, Cambridge, MA) (Kodama and Hu, 2010); for photobleaching experiments, to the monomeric GFP variant GFP(A206K) (Zacharias et al., 2002), kindly provided by Dr Eric Goaux (Vollum Institute, Portland, OR). Ca^2+^-insensitive CaM_1234_ was a gift from Dr Johannes Hell (UC Davis, CA). Mutant rabbit Ca_V_1.2(I1654E), analogous to human I1624E (Zühlke et al., 1999), was used in experiments designed to elucidate the role of the IQ motif in channel interactions. This single point mutation (I1672E) was introduced using the QuikChange II XL Site-Directed Mutagenesis kit (Agilent Technologies, Santa Clara, CA). The role of the pre-IQ motif in channel interactions was examined by exchanging a 33-amino-acid segment, as illustrated in Figure 4—figure supplement 4A. The resultant Ca_V_1.2(pre-IQ swap) was used in whole-cell patch-clamp and bimolecular fluorescence complementation experiments. The general ER marker mCherry-Sec61β was a gift from Gia Voeltz (Addgene plasmid # 49155). Finally, JPH2-GFP was used to tether the ER to the PM in tsA-201 cells.

### Electrophysiology

Ca^2+^ currents were recorded in the whole-cell voltage-clamp or cell-attached patch configurations using borosilicate patch pipettes with resistances of 3–6 μΩ for tsA cells and 2–3 μΩ for cardiomyocytes. Currents were sampled at a frequency of 20 kHz, low-pass–filtered at 2 kHz using an Axopatch 200B amplifier, and acquired using pClamp 10.2 software (Molecular Devices, Sunnyvale, CA). All membrane potentials referred to herein have been corrected for liquid junction potential. All experiments were performed at room temperature (22–25°C).

For whole-cell current recordings from tsA-201 cells, pipettes were filled with a Cs-based internal solution containing 87 mM Cs-aspartate, 20 mM CsCl, 1 mM MgCl_2_, 10 mM HEPES, 10 mM EGTA and 5 mM MgATP, adjusted to pH 7.2 with CsOH. In experiments requiring dialysis of the calmodulin inhibitory peptide MLCKp (EMD Millipore, Darmstadt, Germany; 208735), this chemical was added to the pipette solution. Cells were continuously superfused throughout experiments with our regular external solution containing 5 mM CsCl, 10 mM HEPES, 10 mM glucose, 113 mM NMDG, 1 mM MgCl_2_ and 20 mM CaCl_2_, adjusted to pH 7.4 with HCl. For experiments in which Ba^2+^ was used as the charge carrier in place of Ca^2+^, the perfusate contained 5 mM CsCl, 10 mM HEPES, 10 mM glucose, 140 mM NMDG, 1 mM MgCl_2_ and 2 mM BaCl_2_, adjusted to pH 7.4 with HCl. Current-voltage relationships were obtained by subjecting cells to a series of 300-ms depolarizing pulses from a holding potential of −80 mV to test potentials ranging from −60 to +80 mV. The voltage dependence of conductance was obtained by converting the resultant currents to conductances using the equation, G = *I*_Ca_/[test pulse potential − reversal potential of *I*_Ca_], normalizing (G/Gmax), and plotting conductance vs the test potential.

Cell-attached patch, single-channel currents (i_Ca_) were recorded from tsA-201 cells superfused with high K^+^ solution to fix the membrane potential at ∼0 mV. The solution had the following composition: 145 mM KCl, 2 mM MgCl_2_, 0.1 mM CaCl_2_, 10 mM HEPES and 10 mM glucose; pH was adjusted to 7.3 with KOH. Pipettes were filled with a solution containing 10 mM HEPES and either 110 mM CaCl_2_ or 110 mM BaCl_2_; pH was adjusted to 7.2 with CsOH. The dihydropyridine agonist BayK-8644 (500 nM) was included in the pipette solution to promote longer channel open times. A voltage-step protocol from a holding potential of −80 mV to a depolarized potential of −30 mV was used to elicit currents. The single-channel event-detection algorithm of pClamp 10.2 was used to measure single-channel opening amplitudes and *n*P_o_, and to construct all-points histograms.

To record I_Ca_ from isolated ventricular myocytes, cells were initially perfused with Tyrode's solution. Once the whole-cell configuration was successfully established, the external solution was replaced with one containing 5 mM CsCl, 10 mM HEPES, 10 mM glucose, 140 mM NMDG, 1 mM MgCl_2_ and either 2 mM CaCl_2_ or 2 mM BaCl_2_, adjusted to pH 7.4 with HCl. The pipette was filled with the Cs-based internal solution described above. Facilitation of I_Ca_ was measured using a triple-pulse protocol consisting of two identical test pulses (V_1_ and V_2_), separated by a conditioning pulse to +80 mV (V_pre_), as previously described (Poomvanicha et al., 2011) and illustrated in Figure 7A. The currents elicited by V_1_ and V_2_ were referred to as I_1_ and I_2_, and the ratio between their peaks (I_2_/I_1_) was used as a measure of facilitation.

### Recording of Ca^2+^ sparklets

To measure Ca^2+^ sparklets in tsA-201 cells or ventricular myocytes, cells were patch-clamped in whole-cell mode and held at a hyperpolarized potential of −80 mV to increase the driving force for Ca^2+^ entry. Sub-sarcolemmal Ca^2+^ signals were monitored by dialyzing cells with 200 μM Rhod-2 via the patch pipette and continuously perfusing them with the external solution described above containing 20 mM CaCl_2_ and 10 mM EGTA, a relatively slow Ca^2+^ buffer. With this buffer/indicator combination, Ca^2+^ entering the cell via membrane Ca_v_1.2 channels binds to the relatively fast Ca^2+^ indicator Rhod-2 to generate a fluorescent signal, and the excess EGTA rapidly buffers Ca^2+^, restricting the signal to the point of entry. Sub-sarcolemmal Ca^2+^ signals (Ca^2+^ sparklets) were captured using a through-the-lens TIRF microscope built around an Olympus IX-70 inverted microscope equipped with an oil-immersion ApoN 60×/1.49 NA TIRF objective and an Andor iXON CCD camera. Images were acquired at 100 Hz using TILLvisION imaging software (TILL Photonics, FEI, Hillsboro, OR). Sparklets were detected and analyzed using custom software written in MATLAB (Source code 1). Rhod-2 fluorescence signals were converted to Ca^2+^ concentration units using the F_max_ equation (Maravall et al., 2000). The activity of Ca^2+^ sparklets was determined by calculating the *nP*_s_ of each Ca^2+^ sparklet site, where *n* is the number of quantal levels and *P*_s_ is the probability that a quantal Ca^2+^ sparklet event is active. A detailed description of this analysis can be found in Navedo et al. (Navedo et al., 2005, 2006).

### Coupled Markov chain model

The degree of coupling between single Ca_V_1.2 channels or Ca^2+^ sparklet sites was assessed by further analyzing single-channel and sparklet recordings using a binary coupled Markov chain model (Source code 2), as first described by Chung and Kennedy (1996) and previously employed by our group (Navedo et al., 2010; Cheng et al., 2011; Dixon et al., 2012). The custom program, written in the MATLAB language, assigns a coupling-coefficient (κ) to each record, where κ can range from 0 (purely independently gating channels) to 1 (fully coupled channels). Elementary event amplitudes were set at 0.5 pA for i_Ca_, 1.5 pA for i_Ba_, and 38 nM for Ca^2+^ sparklets.

### Immunocytochemistry and super-resolution microscopy

Transfected tsA-201 cells expressing the relevant Ca_V_1.2 channel constructs were plated onto poly-L-lysine–coated #1.5 coverslips (Thermo Fisher Scientific) the day before fixation. For immunostaining, cells were fixed by incubating for 10 min in ice-cold methanol, then washed with PBS and blocked for 1 hr at room temperature in 50% SEA BLOCK (Thermo Fisher Scientific) and 0.5% vol/vol Triton X-100 in PBS (blocking buffer). The pore-forming subunit of Ca_V_1.2 was probed with a rabbit polyclonal primary antibody (anti-CNC1; kindly provided by Drs William Catterall and Ruth Westenbroek [Hulme et al., 2003]), diluted to 5 μg/ml in diluted blocking buffer (20% SEA BLOCK, 0.5% Triton X-100), by incubating overnight at 4°C. The following morning, cells were washed extensively, receiving three washes with PBS and five washes with diluted blocking buffer. Cells were then incubated for 1 hr at room temperature with Alexa Fluor 647-conjugated donkey anti-rabbit secondary antibody (2 μg/ml; Molecular Probes–Life Technologies) in diluted blocking buffer. Cells were finally washed thoroughly with PBS and mounted for imaging. Native Ca_v_1.2 channels in freshly isolated adult ventricular myocytes were immunostained in an identical manner except that plating procedures differed. Specifically, myocytes were plated onto laminin and poly-L-lysine–coated #1.5 coverslips and allowed to adhere for 1 hr before fixation.

For double-labeling experiments used to examine the co-localization of Ca_V_1.2 channels and the ER, tsA-201 cells transfected with Ca_V_1.2 channels (WT or pre-IQ swap mutants) and mCherry-Sec61β were fixed in 3% paraformaldehyde and 0.1% glutaraldehyde in PBS for 10 min followed by extensive washing in PBS and reduction for 5 min in ∼0.1% sodium borohydride in water to reduce background fluorescence. Cells were washed and blocked as described above and were then incubated in primary antibody solution containing 5 μg/ml rabbit anti-CNC1 and 2 μg/ml rat monoclonal anti-mCherry (Life Technologies) for 1 hr at room temperature. Excess primary antibody was removed by three washes with PBS and five washes with diluted blocking buffer. Cells were then incubated for 1 hr at room temperature with Alexa Fluor 647-conjugated donkey anti-rabbit and Alexa Fluor 568-conjugated chicken anti-rat secondary antibodies (2 μg/ml each; Molecular Probes–Life Technologies) in diluted blocking buffer. Cells were finally washed thoroughly with PBS and mounted for imaging. In some experiments, tsA-201 cells co-expressing JPH2-GFP were fixed and stained as per the double-staining protocol described above. JPH2-GFP was not immunostained, but simply imaged in TIRF mode by exciting the GFP tag.

Coverslips were mounted with MEA-GLOX (for double staining) or β-ME-GLOX imaging buffer on glass depression slides (neoLab, Heidelberg, Germany) and sealed with Twinsil (Picodent, Wipperfürth, Germany). The imaging buffers contained TN buffer (50 mM Tris pH 8.0, 10 mM NaCl), GLOX oxygen scavenging system (0.56 mg/ml glucose oxidase, 34 μg/ml catalase, 10% wt/vol glucose), and either 100 mM MEA (cysteamine) or 142 mM 2-mercaptoethanol (β-ME). Excess imaging buffer was blotted away before application of the Twinsil sealant. This is particularly important with the β-ME-GLOX imaging buffer as the Twinsil will not set if it contacts this buffer.

GSD super-resolution images of Ca_V_1.2 channels in fixed ventricular myocytes or Ca_V_1.2 channels and mCherry-Sec61β in fixed tsA-201 cells were generated using a Leica SR GSD 3D system. The system is built around a Leica DMI6000 B TIRF microscope and is equipped with a Leica oil-immersion HC PL APO 160×/1.43 NA super-resolution objective, four laser lines (405 nm/30 mW, 488 nm/300 mW, 532 nm/500 mW, and 642 nm/500 mW), and an Andor iXon3 EM-CCD. Images were collected in TIRF mode at a frame rate of 100 Hz for 20,000–100,000 frames using Leica Application Suite (LAS AF) software. Ca_V_1.2 cluster area sizes were determined using binary masks of the images in ImageJ/Fiji.

### Stepwise photobleaching

tsA-201 cells expressing Ca_V_1.2 channels tagged at their C-terminus with monomeric GFP were fixed in 4% paraformaldehyde (10 min) and imaged in TIRF mode on the Leica 3D-GSD system described above using a 160×/1.43 NA objective. The core Leica DMI6000 B TIRF microscope in this system is capable of functioning outside of GSD SR imaging mode as a conventional TIRF microscope. Cells were illuminated with 488-nm laser light, and image stacks of 2000 frames were acquired at 30 Hz. The first five images after the shutter was opened were averaged, and a rolling-ball background subtraction was applied using ImageJ/Fiji. This image was then low-pass filtered with a 2-pixel cut-off and high-pass filtered with a 5-pixel cut-off (see Figures 2D and 3D). Thresholding was then applied to identify connected regions of pixels that were above threshold. The ImageJ/Fiji plugin ‘Time Series Analyzer v2.0’ was then used to select 4 × 4 pixel regions of interest (ROIs) centered on the peak pixel in each spot. Next, z-axis intensity profiles (where z is time) from these ROIs were examined over the entire image stack. To facilitate the identification of bleaching steps, the signal-to-noise ratio was improved by applying a 5-pixel rolling-ball background subtraction, a median filter (1 pixel radius), and a 10 frame moving average. Bleaching steps were then manually counted.

Identical procedures were performed on cardiomyocytes expressing photo-activatable, GFP-tagged Ca_V_β_2_ auxiliary subunits. This subunit binds to the pore-forming α_1_ subunit of the channel with a 1:1 stoichiometry; thus, expression of this protein in cardiomyocytes represents a photo-activatable fluorescent marker of Ca_V_1.2 channels. Since adult cardiomyocytes are impervious to chemical transfection, we used adeno-associated virus serotype 9 (AAV9) to transfer this gene into mice via retro-orbital injection, a strategy that has been successfully used by others to transfer cardiac genes in mice (Fang et al., 2012). The AAV9-packaged Ca_V_β_2_-PA-GFP was engineered from Ca_V_β_2_-PA-GFP pcDNA by Vector Biolabs. Mice were sacrificed 5 week after retro-orbital injection. Successful gene transfer was confirmed by photo-activating the Ca_V_β_2_-PA-GFP with 405 nm laser light. Prior to photo-activation, no GFP fluorescence emission was detected upon excitation with 488 nm laser light, but after photo-activation, robust GFP fluorescence emission was observed in the z-lines of isolated ventricular myocytes (data not shown). For stepwise photobleaching experiments, GFP was photo-activated prior to starting movie recordings.

### Bimolecular fluorescence complementation

Spontaneous interactions of Ca_V_1.2 channels were assayed using bimolecular fluorescence complementation. In these experiments, Ca_V_1.2 channels were tagged at their C-terminus with non-fluorescent N- (VN_(1-154, I152L)_) or C-terminal (VC_(155-238, A206K)_) halves of a ‘split’ Venus fluorescent protein. When Ca_V_1.2-VN and Ca_V_1.2-VC are brought close enough together to interact, the full Venus protein can fold into its functional, fluorescent confirmation. The magnitude of Venus fluorescence emission therefore provides an indicator of Ca_V_1.2 interactions. Venus fluorescence was monitored in tsA-201 cells expressing Ca_V_1.2-VN and Ca_V_1.2-VC using TIRF microscopy, as described above (‘recording of Ca^2+^ sparklets’). Transfected cells were identified by co-expression of tagRFP or, in experiments requiring Ca^2+^ imaging with Rhod-2, by weak initial Venus expression.

The relationship between membrane voltage and Ca_V_1.2 interactions was obtained by subjecting patch-clamped cells in whole-cell mode to a series of 9-s depolarizations from a holding potential of −80 mV to test potentials ranging from −60 to +80 mV. Maturation of newly reconstituted Venus protein takes some time, hence the long depolarizing pulse (Nagai et al., 2002). Cells were superfused throughout with the 20 mM Ca^2+^ external solution described above. A TTL pulse generated by the TILL imaging system was used to trigger the onset of each voltage sweep. The cell TIRF footprint was illuminated using 491-nm light throughout each voltage sweep, and PM-localized Venus fluorescence emission was monitored in a TIRF movie acquired at a rate of 100 Hz. The final six frames of each movie were averaged to generate a single image for each voltage sweep. These images were median filtered (1 pixel radius) then divided by the −60 mV image to obtain Venus F/F_0_ images. Finally, the images were smoothed and pseudo-colored using the ‘red hot’ lookup table in ImageJ/Fiji. The Ca^2+^ dependence of Ca_V_1.2 interactions was tested by performing the aforementioned procedure first with 2 mM Ba^2+^ as the conducting ion, then switching the perfusate to our regular external solution described above (20 mM Ca^2+^) and running the procedure again on the same cell (see Figure 4—figure supplement 2).

Reconstitution of the Venus protein is irreversible; thus, once channels spontaneously interact, they remain fused together. We exploited this feature of the bimolecular fluorescence complementation assay in experiments designed to investigate the physiological effects of Ca_V_1.2 interactions. In these experiments, transfected tsA-201 cells expressing the split-Venus–tagged channels were patch-clamped in whole-cell mode and dialyzed with 200 μM Rhod-2 via the pipette. Ca^2+^ sparklets were recorded while holding the cell at −80 mV. The depolarizing step protocol described above was performed, and Venus fluorescence was monitored as before. Increases in Venus fluorescence emission during the protocol provide an indication of Ca_v_1.2 interactions. Holding cells once more at −80 mV, Ca^2+^ sparklet activity was recorded from the irreversibly fused Ca_V_1.2 channels.

### Dynamic FRET measurements in live cells

For this set of experiments, tsA-201 cells were transfected with expression constructs for Ca_V_1.2-EGFP and Ca_V_1.2-tagRFP. FRET from Ca_V_1.2-EGFP (donor) to Ca_V_1.2-tagRFP (acceptor) was measured on an Olympus Fluoview 1000 (FV1000) confocal laser-scanning microscope equipped with an Olympus APON 60×/1.49 NA oil-immersion objective. A 473-nm diode laser was used to excite the sample. Emitted light was separated with an SDM560 beam splitter, collected with BA490-540 and BA575-675 emission filters, and detected by a photomultiplier tube. The resultant raw donor and acceptor images were corrected for background and bleed-through of GFP emission into the RFP channel. In separate experiments using cells expressing only Ca_V_1.2-tagRFP, bleed-through was determined to be ∼16%. Intensity measurements from the corrected images, referred to as GFPg and RFPg respectively (where g refers to excitation of GFP with 473 nm light), were extracted using Metamorph or ImageJ software. FRET was expressed as the ratio, FRETr = RFPg/GFPg. In some experiments, the time course of FRETr was monitored during photolysis of caged Ca^2+^. In others, the time course of FRETr was recorded simultaneously with whole-cell Ca_V_1.2 currents. A TTL pulse generated by the FV1000 system at the onset of imaging was used to trigger a voltage protocol consisting of a 500-ms depolarizing pulse from a holding potential of −80 to +10 mV. Finally, the Ca^2+^ dependence of FRET between Ca_V_1.2 channels was monitored while perfusing cells with external solutions containing 5 mM CsCl, 10 mM HEPES, 10 mM glucose, 140 mM NMDG, 1 mM MgCl_2_ and increasing concentrations (0, 25, 50, 100, 200, 300, 400, 800 and 5000 nM) of CaCl_2_. The solutions were adjusted to pH 7.4, and 1 μM ionomycin was added to equilibrate intracellular and extracellular Ca^2+^.

### Photolysis of caged Ca^2+^

tsA cells were perfused with a ‘zero calcium’ external solution containing 5 mM CsCl, 10 mM HEPES, 10 mM glucose, 140 mM NMDG, 1 mM MgCl_2_, and 2 mM BaCl_2_ (pH. 7.4). Cells were patch-clamped in whole-cell mode as previously described (Tadross et al., 2013) using an internal solution containing 5 mM CsCl, 40 mM HEPES, 135 mM CsMeSO_3_, 1 mM citrate and 1.6 mM CaCl_2_, adjusted to pH 7.4 and frozen into aliquots. On the day of experiments, the Ca^2+^ cage DMNP-EDTA (2 mM; Invitrogen, D6814) was added. Mg^2+^ was omitted from the pipette solution since DMNP-EDTA can also cage Mg^2+^. Internal solutions were supplemented with 0.25 μM PI(4,5)P_2_ (Avanti Polar Lipids, Alabaster, AL; 840046P) and 0.5 μM okadaic acid (LC Laboratories, Woburn, MA; O-2220) to stabilize I_Ca_ (Tadross et al., 2013). This enabled us to record stable currents and achieve successful uncaging of Ca^2+^, as assayed with the Ca^2+^-sensitive dyes, Fluo-4 AM or Rhod-2 AM (see Figure 6A and Figure 4—figure supplement 1). Uncaging experiments were performed on an Olympus FV1000 confocal microscope equipped with a SIM scanner unit that permits simultaneous laser light uncaging (100% 405 nm for two frames) and recording of Venus, Fluo-4, Rhod-2 or EGFP/tagRFP FRET fluorescence emission.

### CaMKII activity assay

CaMKII activity was measured using the ‘SignaTECT Calcium/Calmodulin-Dependent Protein Kinase Assay System’ (Promega Corporation, Madison, WI). Mouse hearts were homogenized in a lysis buffer solution containing 20 mM Tris–HCl (pH 8.0), 2 mM EDTA, 2 mM EGTA, 2 mM DTT, PhosSTOP phosphatase inhibitor cocktail (Roche Diagnostics GmbH, Mannheim, Germany) and cOmplete, Mini protease inhibitor cocktail (Roche Diagnostics). Hearts were subjected to three 5 s pulses at 12,000–17,000 rpm using a PRO200 Homogenizer Unit (Pro Scientific, Oxford, CT). The homogenate was centrifuged at 2000 rpm for 10 min and the resultant supernatant was collected and assayed for CaMKII activity as per the manufacturers instructions. To determine the effect of MLCKp on CaMKII activity, 0.1 or 1 μM MLCKp was added to the reaction.

## Additional files

Source code 1. Custom software for Ca^2+^ sparklet detection and analysis written in MATLAB. **DOI:** http://dx.doi.org/10.7554/eLife.05608.019

Source code 2. Binary coupled Markov chain model. **DOI:** http://dx.doi.org/10.7554/eLife.05608.020
